# SCoTCH-seq reveals that 5-hydroxymethylcytosine encodes regulatory information across DNA strands

**DOI:** 10.1073/pnas.2512204122

**Published:** 2025-07-31

**Authors:** Jack S. Hardwick, Somdutta Dhir, Angie Kirchner, Angela Simeone, Sean M. Flynn, James M. Edgerton, Rafael de Cesaris Araujo Tavares, Isabel Esain-Garcia, David Tannahill, Paula Golder, Jack M. Monahan, Walraj S. Gosal, Shankar Balasubramanian

**Affiliations:** ^a^Yusuf Hamied Department of Chemistry, University of Cambridge, Cambridge CB2 1EW, United Kingdom; ^b^Cancer Research UK Cambridge institute, University of Cambridge, Cambridge CB2 0RE, United Kingdom; ^c^biomodal Ltd., The Trinity Building, Cambridge CB10 1TS, United Kingdom; ^d^School of Clinical Medicine, University of Cambridge, Cambridge CB2 0SP, United Kingdom

**Keywords:** 5-hydroxymethylcytosine, 5-methylcytosine, epigenetics, DNA sequencing

## Abstract

In mammalian genomes, cytosine modifications form a layer of regulatory information alongside the genetic code. Decoding this information is crucial to our understanding of biology and disease. Established sequencing methods cannot simultaneously resolve cytosine’s three most common forms—cytosine (C), 5-methylcytosine (mC), and 5-hydroxymethylcytosine (hmC)—across both strands of the DNA double helix. Thus, how epigenetic information is distributed in DNA remains unclear. Here, we present *S*trand-*Co*upled *T*andem *C*ytosine *H*ydroxymethylation and methylation sequencing (SCoTCH-seq): an accurate and quantitative, base-resolution approach to sequence genomes, together with mC and hmC, in both strands of the same DNA fragment. We show that different forms of cytosine combine across the double helix at CpG sites to form discrete information states in the mouse epigenome. These CpG states have distinct genomic distributions—including at promoters, enhancers, and gene bodies—and have different relationships with transcription. We show that while all possible forms of hydroxymethylation occur, hmC is predominantly asymmetric and that different forms of asymmetric hmC are not equivalent. Our findings demonstrate that 5-hydroxymethylcytosine combines with different cytosine variants across the DNA double helix to form distinct states of regulatory information.

## Significance

Modified cytosine bases are essential components of mammalian genomes, but how they are distributed in double-stranded DNA remains unclear. Here, we report a method to simultaneously sequence the two most abundant modified bases—5-methylcytosine and 5-hydroxymethylcytosine (hmC)—across both strands of genomic DNA. Applying this method to DNA from mouse embryonic stem cells, we find that at CpG sites, hmC forms different combinations with cytosine variants across the DNA double helix, exhibiting unique distributions at enhancers and gene bodies, and distinct relationships with transcription. Our findings demonstrate that the double-stranded context of hmC is a key feature of the epigenome, revealing a further layer of regulatory information.

Each of us started life as a single cell, with a single set of genetic instructions. During development, these same instructions are used selectively to create every other cell in the body. This remarkable feat is achieved by epigenetic gene regulation—the specific control of gene expression that does not relate to changes in DNA sequence. In mammals, cytosine methylation plays a critical role in epigenetic gene regulation, maintaining cell identity through its involvement in stable transcriptional repression (1). While 5-methylcytosine (mC) deposited during embryogenesis can persist throughout one’s entire lifetime (1), it can also be converted to 5-hydroxymethylcytosine (hmC) by the Ten-eleven translocation (Tet) family of enzymes (2). This can lead to demethylation by either a replication-dependent (passive) or independent (active) pathway, where hmC is further oxidized to 5-formylcytosine (fC) or 5-carboxylcytosine (caC) before being excised and replaced with unmodified cytosine (C) (3).

Beyond being a transient demethylation intermediate, hmC can itself be a detectable, stable mark (4, 5). In contrast to its oxidized derivatives, hmC cannot be directly removed from the genome (6), and its abundance is orders of magnitude greater across different cell types (3). Hydroxymethylation at gene bodies positively correlates with transcription level (7–10) and is associated with maintaining transcriptional fidelity in multiple cell types (11, 12). The mechanistic basis of these findings remains unclear, but hmC may play a causal role (10–12). Notably, hmC distribution is highly cell-type specific (7, 8), and its dysregulation is linked to diseases ranging from Alzheimer’s (13) to cancer (6, 14)—where a global depletion of hmC has been observed in virtually every tumor type tested (6). Consequently, hmC signatures in circulating cell-free DNA constitute a clinically promising biomarker (14). Elucidating the distribution and role of hmC, and its complex interplay with mC, is thus essential to our understanding of fundamental biology and disease.

**Fig. 1.**
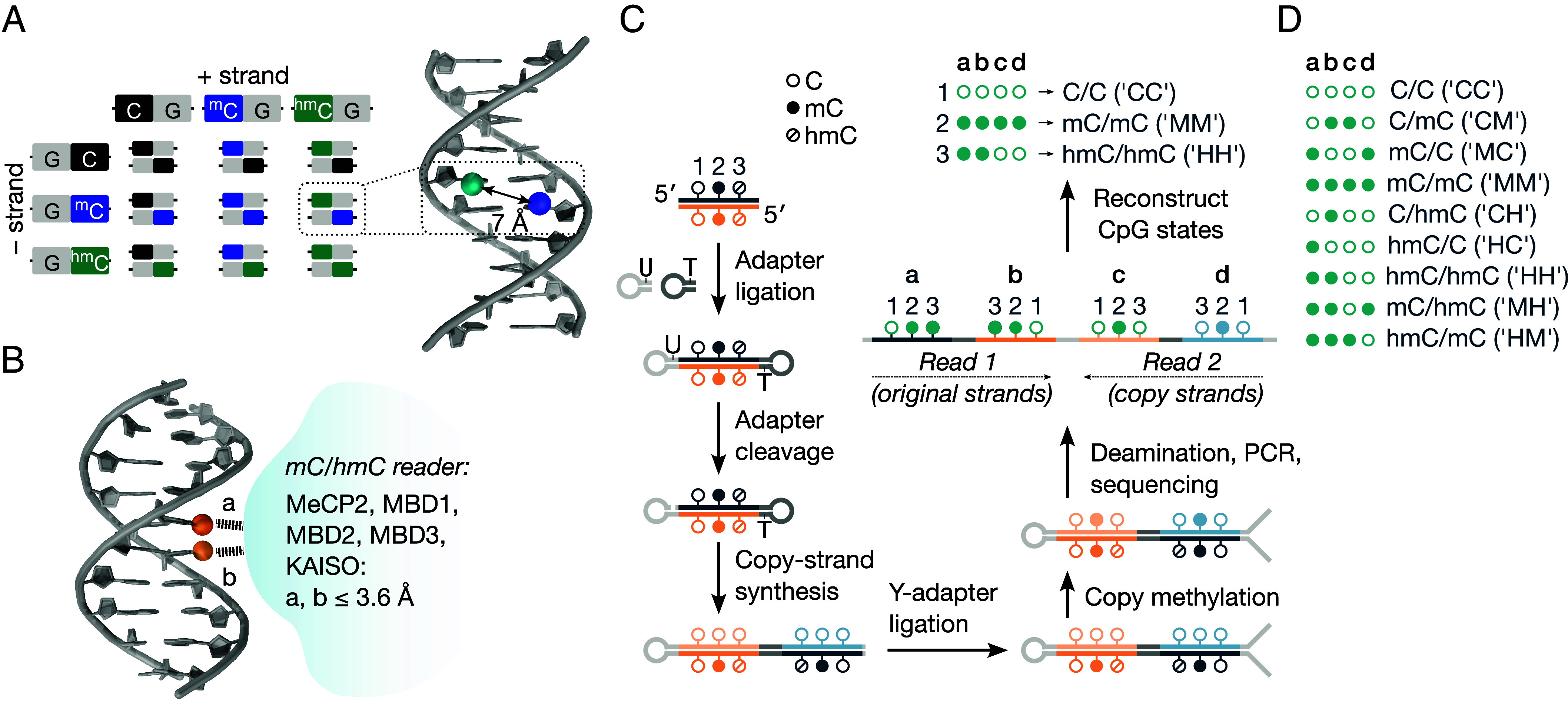
Quantitative sequencing of C, mC, and hmC in double-stranded DNA. (*A*) CpG sites contain a cytosine residue on each DNA strand, resulting in nine possible combinations of C, mC, and hmC (21). Within an individual CpG site, both cytosine modification sites are close (~7 Å, *SI Appendix*, Fig. S1). Each CpG state thus produces a unique structural signature in the major groove for protein recognition, and many readers of cytosine modifications interrogate both modification sites simultaneously (*SI Appendix*, Fig. S1). (*B*) Schematic of several known readers of modified cytosines that interact simultaneously with the modifications on both strands of a CpG site, forming close contacts (“a”, “b” ≤ 3.6 Å, see *SI Appendix*, Fig. S1 for full analysis). (*C*) Schematic of experimental workflow, which enables all nine CpG states to be sequenced at single-base resolution from a single sample. Sonicated, end-repaired DNA fragments are ligated to a 1:1 mixture of hairpin adapters. Uracil excision from one adapter forms a self-priming construct, which is extended by Klenow *exo–* polymerase to generate copy strands that complement each original DNA strand, followed by ligation to an Illumina-compatible Y-adapter. Base conversion steps follow the method of ref. 22. Briefly, copy strands are copy-methylated with a high-fidelity methyltransferase (DNMT5) only at CpG sites that contain mC on the original strands, whereas a prior glucosylation step prevents copying at hmC. mC is then protected by TET2 oxidation and glucosylation, and unmodified cytosines are enzymatically deaminated (APOBEC3A and UvrD helicase), disrupting the hairpin structure and enabling efficient amplification and Illumina sequencing. Both original DNA strands are sequenced in Read 1, their copy strands in Read 2. The complete modification status of each original CpG site (numbered 1 to 3 above) is encoded by four corresponding methylation calls, which are extracted with a custom bioinformatics pipeline. (*D*) The four-digit methylation calls encode all nine possible combinations of C, mC, and hmC. Standard B-DNA models in *A* and *B* were generated using w3DNA (23).

mC and hmC occur most frequently at CpG sites (15), a palindromic dinucleotide sequence with a cytosine residue on each DNA strand. A CpG site can comprise nine possible combinations of C, mC, and hmC, which we call “CpG states” (Fig. 1*A*). Within a CpG site, the modification sites of the two cytosines are close in space (~7 Å) and occupy the major groove (Fig. 1*A*). Thus, each CpG state presents a unique structural arrangement for protein recognition. Indeed, crystal structures of many known mC and hmC reader proteins show that they directly interact with both modification sites across the DNA strands (Fig. 1*B* and *SI Appendix*, Fig. S1). For example, MeCP2, which interacts with both mC and hmC and plays a critical role in the brain (16), is sensitive to the double-stranded form of hmC (17). Readers of hemimethylated CpG sites in eukaryotes are also known, such as CDC7A (18, 19), UHRF1 (17), and the plant methyltransferase MET1 (20), which demonstrates proteins’ ability to discriminate between different CpG states. But how these epigenetic cytosine states are distributed in the double-stranded epigenome remains poorly understood. This is due to limitations in conventional sequencing approaches that have prevented the quantitative, simultaneous sequencing of C, mC, and hmC across both DNA strands. Here, we introduce *S*trand-*Co*upled *T*andem *C*ytosine *H*ydroxymethylation and methylation sequencing (SCoTCH-seq)—an accurate, quantitative method for simultaneously sequencing C, mC, and hmC in double-stranded DNA that reveals such CpG states.

## Results

### Quantitative, Simultaneous Sequencing of C, mC, and hmC in Double-Stranded DNA.

To determine the double-stranded epigenetic states comprising C, mC, and hmC, we have developed a sequencing approach that quantitatively resolves all three cytosine states simultaneously on both strands of the DNA double helix. In this method, the two strands of each DNA fragment are physically tethered throughout the experiment by a hairpin adapter (Fig. 1*C*). This prevents separation of the strands and the loss of duplex information that would otherwise occur (24). Unmodified copy strands of both original DNA strands are then produced within each construct. A series of enzymatic conversions of the resulting constructs enables C, mC, and hmC on each strand to be sequenced at single-base resolution, adapting our recently published method to simultaneously sequence C, mC, and hmC in single-stranded DNA (22).

In the SCoTCH-seq workflow, genomic DNA is first fragmented by sonication. Fragments of ~100 bp are then size-selected by automated pulsed-field electrophoresis. These fragments are 5’-phosphorylated and 3’-A-tailed, followed by ligation to synthetic hairpin adapters. In contrast to our previous work (22), which uses a single, cleavable hairpin (containing 2’-deoxyuridine, dU), this method uses a 1:1 mixture of two different hairpin adapters—one that is cleavable (containing dU) and one that is not (containing only canonical nucleotides). Target constructs are capped with each of the hairpin adapters. The dU from the adapter at one end of the construct is enzymatically cleaved. The resulting self-priming construct is extended by Klenow *exo*–, forming a larger hairpin in which the two original DNA strands are paired with newly synthesized “copy” strands (Fig. 1*C*). Note that side products form during ligation of the hairpin adapters that can contain two cleavable or two noncleavable adapters. During copy-strand formation, these side products do not change in size, whereas the target constructs roughly double in length, enabling their enrichment by size selection. Target constructs are then ligated to a standard, Illumina-compatible adapter. At this stage, CpG sites may be i) fully unmodified on both strands, ii) hemimethylated, or iii) hemihydroxymethylated (protected by glucosylation). For subsequent base-conversion steps, we use the established method described previously (22). Briefly, DNMT5-mediated copy-methylation converts hemimethylated CpG to symmetrically methylated CpG sites but leaves other CpG sites (containing either C or hmC) unchanged. Next, mC and hmC are protected from deamination by TET2 oxidation and glucosylation, and all unmodified cytosines are then deaminated using APOBEC3A and UvrD helicase. The resulting loss of complementarity within the hairpin enables efficient PCR amplification and Illumina sequencing. Both original strands of the DNA fragment are sequenced in Read 1, their copy strands in Read 2. Thus, each CpG site has four associated cytosines, whose methylation calls are extracted by a custom bioinformatics pipeline. In this pipeline, target reads are selected based on their hairpin-adapter content—containing sequences corresponding to both the cleavable and noncleavable adapters in a defined pattern (Fig. 1*C*). By contrast, side products (e.g., those containing only single-stranded information, which constitute on average 16.5% of raw reads) do not contain this configuration of hairpin-adapter sequences and are filtered out. Next, the four insert sequences are extracted from flanking hairpin sequences. The two resulting pairs (a & d, and b & c in Fig. 1*C*) of complementary sequences are aligned in paired-end mode, followed by deduplication and methylation calling. Each CpG site (numbered 1 to 3 in Fig. 1*C*) in an original double-stranded fragment is thus described by four methylation calls, which encode all nine possible CpG states comprising C, mC, and hmC (Fig. 1*D*). Seven other combinations of methylation calls are also possible but do not correspond to plausible CpG states, so these rarely observed calls (0.31% of all CpG calls) are discarded downstream. Note that this workflow may be used with any other sequencing technology that distinguishes between canonical DNA bases by substituting the Illumina-compatible adapter. This method cannot be used in its current form for 5-formylcytosine or 5-carboxylcytosine, whose signal would in any case be negligible in the case studied due to their low abundance in mESCs and other tissue (≤ 0.00001% per dG) (3).

**Fig. 2.**
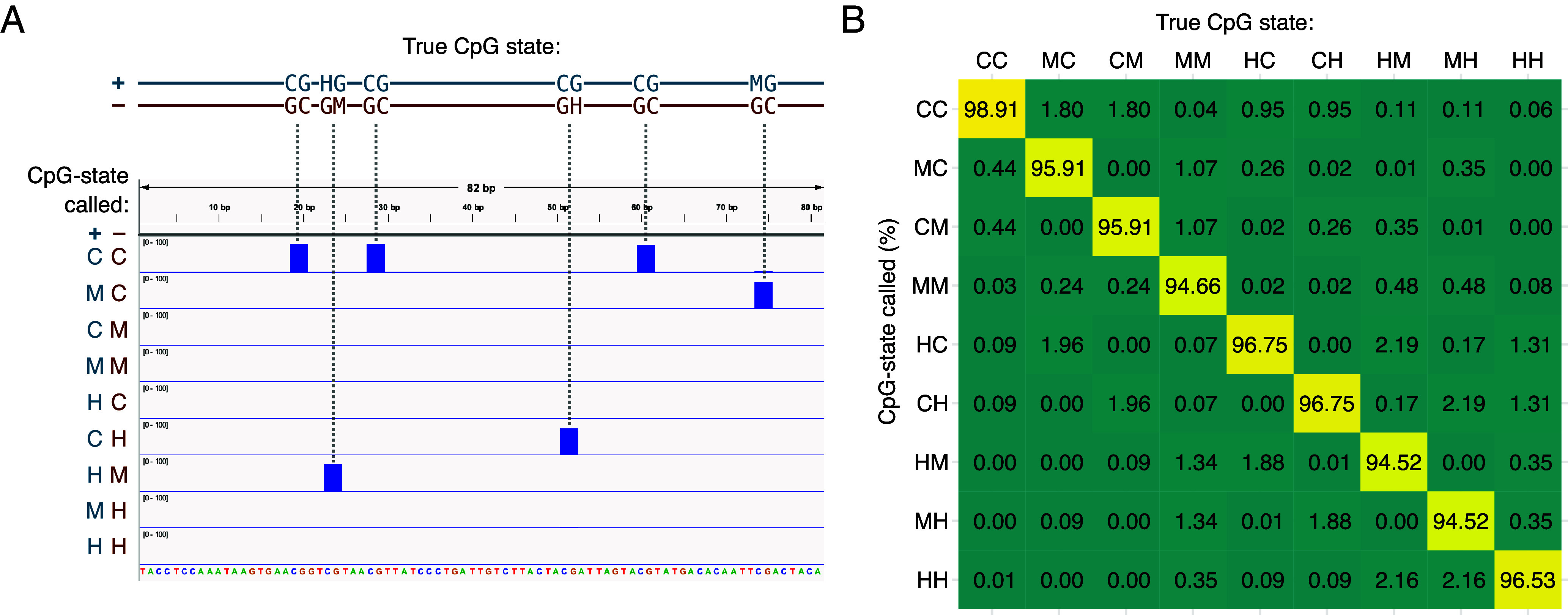
All nine CpG states are accurately resolved in a single experiment. (*A*) Synthetic spike-in containing known CpG states (double-stranded combinations of C, mC, and hmC at CpG sites) with its corresponding CpG-state readout, viewed in IGV (26). Each CpG state is correctly and unambiguously called. (*B*) Confusion matrix showing the rate at which each CpG state in the synthetic spike-ins was correctly called (yellow) versus the rate it was miscalled as each other state (green). All states are called with an accuracy exceeding 94%.

We performed SCoTCH-seq on genomic DNA isolated from two different passages of E14 mouse-embryonic stem cells (mESCs) in the primed state (serum/LIF conditions). E14 mESCs are a well-studied model system with a wealth of genomic, epigenetic, and transcriptomic datasets available (25). To quantify the accuracy of our method, synthetic control oligonucleotide duplexes containing all possible permutations of CpG states comprising C, mC, and hmC (Fig. 2*A* and *SI Appendix*, Table S1) were added to the sonicated samples following size selection, along with fully CpG-methylated bacteriophage l DNA, and unmethylated pUC19. We sequenced the two independent replicates on a NovaSeq 6000 (SP flow cell, 500 cycles), producing an average of 762 million indexed, paired-end reads per run. The average PCR and cluster duplication rate was 12.2%. Evaluation of the spike-in controls showed that our method accurately resolves all nine CpG states comprising C, mC, and hmC, with a call-rate accuracy of over 94% in each case (Fig. 2*B*). Call-rate accuracies (Pearson’s *R* = 0.999, *P* < 2.2 × 10^−16^) and bioinformatics analysis (*SI Appendix*, Figs. S2–S5) show close agreement between the two independent datasets. The E14 datasets were thus merged to maximize depth and coverage, resulting in 94.4% of the genome being covered by at least one read—including 18.7 million CpG sites at an average depth of 12.9×. Because both original DNA strands are covered in each read pair, this corresponds to a depth of 25.8× when compared to conventional, single-stranded methods. Following additional filtering steps (see Methods), 15.8 million CpG sites were retained.

### hmC in mESCs Exists in All Forms and is Mostly Asymmetric.

**Fig. 3.**
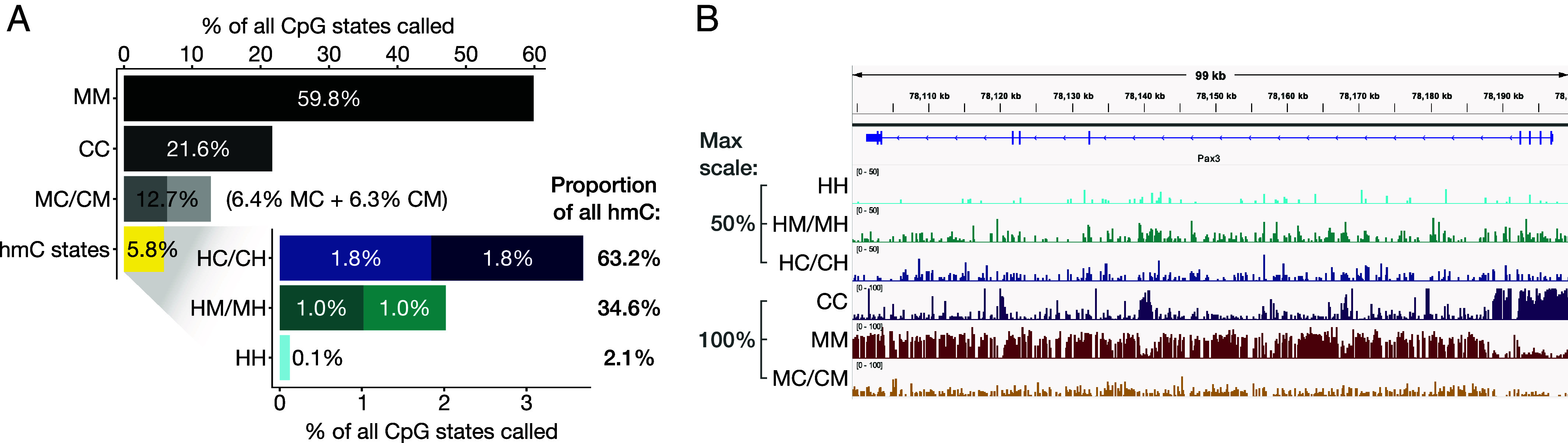
Global abundance and local distribution of CpG states. (*A*) Genome-wide abundance of the nine CpG states comprising C, mC, and hmC, whose distributions depend strongly on their double-stranded context. 18% of all CpG sites are asymmetrically modified. This includes 98% of all hydroxymethylated sites, where, in addition to hmC, the CpG site contains either C (denoted “HC” and “CH,” with hmC on the plus and minus strand, respectively) or mC (“HM” and “MH”). (*B*) Illustrative example of the local distribution of CpG states in the bivalent *Pax3* gene, which encodes a transcription factor critical during embryogenesis and whose aberrant expression is linked to several forms of cancer (27). Data are scaled from 0 to 100%, except for hmC states which are scaled from 0 to 50% for clarity [viewed in IGV (26)].

**Fig. 4.**
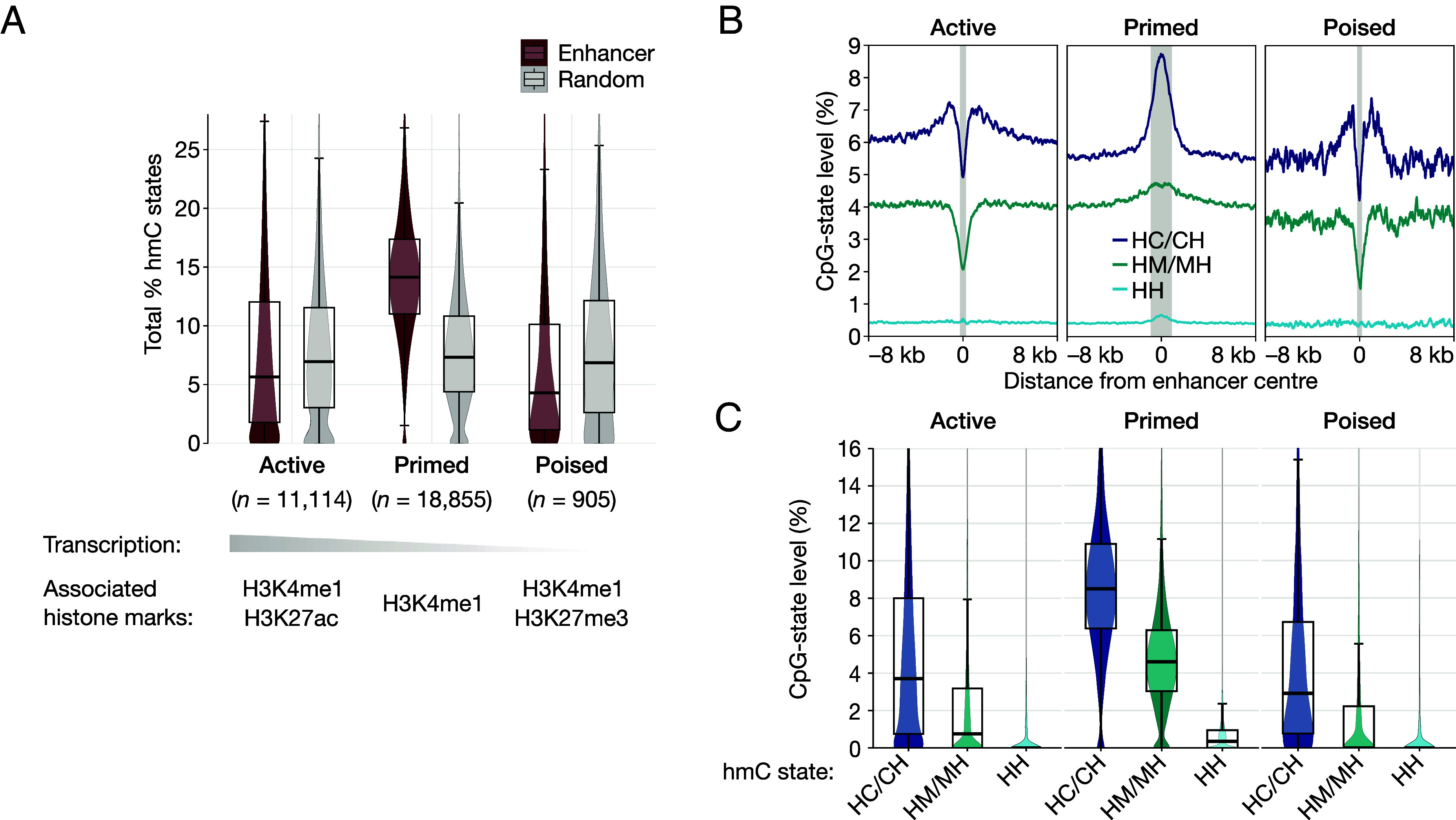
Distribution of hmC states at active, primed, and poised enhancers. (*A*) Total levels of hmC across each enhancer type (crimson), alongside background hmC levels (gray, randomly selected genomic regions of the same size). For each enhancer type—active (*n* = 11,114), primed (*n* = 18,855), and poised (*n* = 905)—characteristic features are also shown (28). Levels are the mean percentage of hydroxymethylated CpG states for each enhancer. Primed enhancers contain high levels of hmC, whereas at active and poised enhancers hmC is somewhat depleted. (*B*) Distribution of hmC states throughout enhancers (200-bp bin size). Shaded regions correspond to the mean length of each enhancer type. Primed enhancers are characterized by a sharp increase in HC/CH and a modest increase in HM/MH, with levels highest at the center of enhancers. Conversely, in active and poised enhancers levels of HC/CH and HM/MH are lowest at the center, though HC/CH levels are elevated at regions directly flanking the enhancers. (*C*) Mean levels of hmC states across each enhancer. hmC at enhancers is overwhelmingly asymmetric and is almost entirely in the HC/CH context, except at primed enhancers, which also contain substantial but lower levels of HM/MH. Median levels of HC/CH at primed enhancers are more than double that at active and poised enhancers. Box-plots: center line, median; box limits, upper and lower quartiles; whiskers, 1.5× interquartile range.

**Fig. 5.**
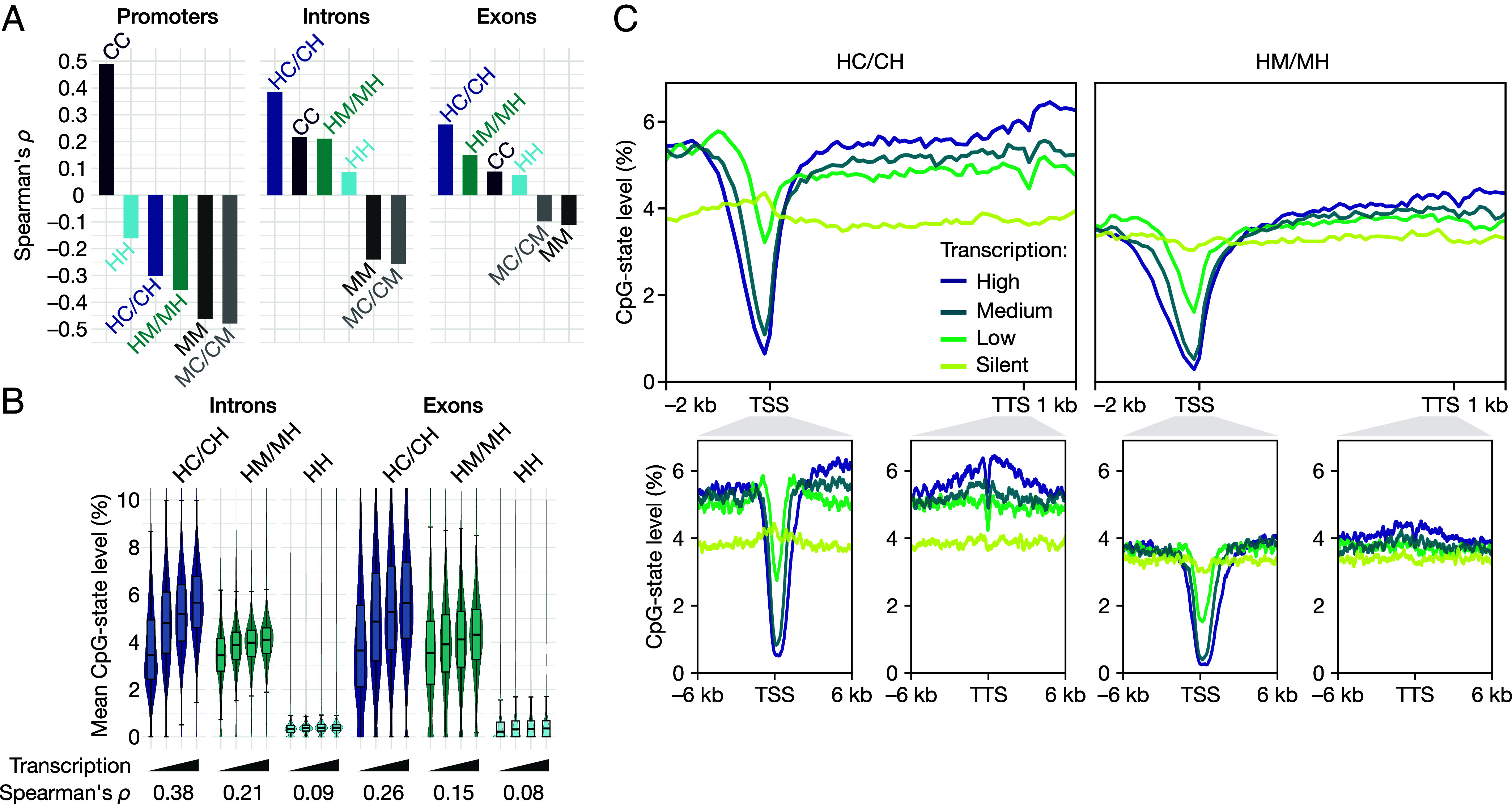
hmC in the HC/CH context dominates hmC’s relationship with transcription at gene bodies and marks transcriptionally active regions. (*A*) Spearman’s correlation coefficients between transcription and CpG-state levels at promoters, introns, and exons of protein-coding genes (*P* < 2.2 × 10^−16^ in all cases, *n* = 16,357, 15,564, and 15,265 genes, respectively). (*B*) Mean levels of hmC states across introns and exons of protein-coding genes, grouped by transcription level: silent (*n* = 4,271), low (*n* = 3,458), medium (*n* = 4,004), and high (*n* = 4,258). Intronic HC/CH levels positively correlate with transcription, with a magnitude close to promoter methylation (*ρ* = 0.38, *P* < 2.2 × 10^−16^ in all cases). The other hmC states, HM/MH and HH exhibit much weaker, positive correlations. (*C*) Distributions of HC/CH and HM/MH across protein-coding genes grouped by transcription level: silent (yellow, *n* = 6,306), low (light green, *n* = 3,778), medium (dark green, *n* = 4,276), and high (blue, *n* = 4,485). In silent genes, the highest levels of HC/CH occur at the TSS, where there is a sharp inversion even for lowly transcribed genes. For all active genes, HC/CH levels are considerably higher than HM/MH levels either side of the TSS region, around the TTS, throughout gene bodies, and even several kilobases upstream of promoters. Box-plots: center line, median; box limits, upper and lower quartiles; whiskers, 1.5× interquartile range.

Having established the accuracy of our method, we performed a global analysis of the mESC epigenome, correcting for the low levels of false positive and negative rates associated with each CpG-state call, as determined from the spike-in data. Our data reveal that all nine possible CpG states comprising C, mC, and hmC exist at substantial levels in the mESC epigenome. Symmetric methylation (MM) is the most common state globally (60% of all CpG state calls), followed by symmetric unmodified CpG (CC, 22%, Fig. 3*A*). The next most abundant states are the two forms of hemimethylation (MC and CM denoting mC on the plus and minus strands, respectively), which together constitute 13% of all CpG states. The remaining 6% of CpG states are hydroxymethylated. Strikingly, the vast majority (98%) of hmC is asymmetric, with hmC on one DNA strand and either C or mC on the other (denoted HC/CH and HM/MH, respectively). Only 2% of all hydroxymethylated CpG sites are symmetrically modified, constituting just 0.1% of all CpG states. The two forms of asymmetric hmC (HC/CH and HM/MH) occur at different rates, with levels of the HC/CH states being almost double those of HM/MH (3.6% and 2% of all CpG states, respectively, Fig. 3*A*). The genomic distributions of different hmC states are distinct (Figs. 3–5 and *SI Appendix*, Figs. S3, S5, and S6).

### Primed Enhancers are Characterized by High Levels of HC/CH.

Enhancers are genetic elements that regulate the transcription of distal, cognate genes. They play a central role in cell differentiation by establishing cell-type-specific gene expression programs (29). Oxidation of mC to hmC is key to modulating enhancer activity during the early stages of differentiation (30), with enhancers exhibiting among the highest enrichment of hmC in every cell type examined (31). We determined the distribution of all nine CpG states comprising C, mC, and hmC, at active, primed, and poised enhancers, using the enhancer classifications of Cruz-Molina et al. (28) (whose primary characteristics are outlined in Fig. 4*A*). Contrary to previous reports (15, 30) we find that hmC at active and poised enhancers is somewhat lower than at randomly sampled regions of the genome (Fig. 4*A*). Primed enhancers, however, contain much higher levels of hmC than do other enhancer types and randomly sampled regions (Fig. 4*A*). We next looked at how individual hmC states are distributed around enhancers. The HC/CH states are the most abundant form of hmC across all enhancer types, followed by HM/MH (Fig. 4 *B* and *C*). Notably, primed enhancers exhibit a sharp increase in HC/CH relative to background levels (Fig. 4*B*, S3). By contrast, we observe only a small increase in the HM/MH and HH states at these loci. Unlike primed enhancers, the centers of active and poised enhancers exhibit the lowest levels of both HC/CH and HM/MH. Levels of HC/CH, however, are elevated at the flanking regions of active and poised enhancers, relative to background levels (Fig. 4*B*). The HM/MH states do not exhibit this trend, with levels depleted throughout active and poised enhancers (Fig. 4 *B* and *C* and *SI Appendix*, Fig. S3).

To gain further insight into the role of hmC at enhancers, we reanalyzed published whole-genome bisulfite sequencing data from Tet-triple knockout (TKO) mESCs that lack Tet1, Tet2, and Tet3 (32). Our results suggest that hmC at primed enhancers is primarily not a demethylation intermediate. Despite primed enhancers containing by far the highest hmC levels, Tet-mediated demethylation at these enhancers is considerably lower than at active or poised enhancers (*SI Appendix*, Fig. S7). These findings point to a functional role of hmC at primed enhancers that extends beyond demethylation.

Primed enhancers also contain much higher methylation levels (both hemi- and symmetric) than poised and active enhancers—exceeding those of unmodified cytosine (*SI Appendix*, Fig. S6). Overall, our findings reveal a unique relationship between hydroxymethylation, methylation, and primed enhancers and that the distribution of hmC at enhancers strongly depends on its double-stranded form.

### hmC in the HC/CH Context Marks Active Genes.

Along with enhancers, gene bodies of the most highly expressed genes are enriched in hmC across mammalian cell types (31). Gene-body hmC positively correlates with transcription across a broad range of mammalian tissues (7–9) and relates to maintaining transcriptional fidelity in early mouse embryogenesis (11) and smooth muscle cells (12). The molecular basis of these relationships remains to be fully established, but hmC is suggested to play a causal role (10–12). Strikingly, our results reveal that the relationship between hmC and transcription strongly depends on its double-stranded context (HC/CH, HM/MH, or HH). First, we determined the distributions of hmC states at gene bodies, transcription start sites (TSSs) and transcription termination sites (TTSs) for protein-coding genes grouped by transcript level. All hmC states exhibit unique distributions, with HC/CH showing the strongest relationship with transcription (Fig. 5). At gene bodies, HC/CH levels increase substantially with increasing transcription (Fig. 5). This effect is much stronger than for HM/MH (Fig. 5) and is negligible for HH (Fig. 5*A*). HC/CH levels also increase markedly with transcription around the TTS—the highest levels occurring just downstream of the most highly transcribed genes (Fig. 5*C*). By contrast, at the TSS we observe an inverse relationship between transcription and HC/CH levels. Within silent genes, HC/CH levels at the TSS are higher than in any other region, whereas within active genes they are at their minimum, decreasing with increasing transcriptional activity (Fig. 5*C*). Notably, even several kilobases upstream and downstream of the TSS and TTS, HC/CH levels differ considerably according to transcript level, while HM/MH levels do not (Fig. 5*C*).

Next, we determined the correlation between the levels of each CpG state and transcription at promoters (±1 kb from the TSS), introns, and exons (Fig. 5*A*). We find that this relationship is strongly affected by both the CpG state and its genomic context. At promoters, all modified CpG states negatively correlate with transcription. This relationship is strongest for hemimethylation (*ρ* = –0.48, *P* < 2.2 × 10^–16^), closely followed by symmetric methylation (*ρ* = –0.46, *P* < 2.2 × 10^–16^). Of the hydroxymethylated states, HM/MH exhibits the strongest relationship at promoters (*ρ* = –0.35, *P* < 2.2 × 10^–16^), followed by HC/CH (*ρ* = –0.30, *P* < 2.2 × 10^–16^) and HH (*ρ* = –0.16, *P* < 2.2 × 10^–16^). At promoters, the unmodified CC state is the only CpG state that correlates positively with transcription, its magnitude being comparable to hemi- and symmetric methylation (*ρ* = 0.49, *P* < 2.2 × 10^–16^).

At gene bodies, by contrast, our results reveal that the relationship between all hmC states and transcription is positive in all cases. Furthermore, HC/CH exhibits the strongest correlation with transcription of any CpG state (Fig. 5 *A* and *B*). Notably, the strength of this relationship (*ρ* = 0.38, *P* < 2.2 × 10^–16^, Fig. 5*A*) is comparable to that of symmetric methylation at promoters (*ρ* = –0.46, *P* < 2.2 × 10^–16^). We find that gene-body methylation (hemi- and symmetric) negatively correlates with transcription. The two forms of methylation exhibit similar strengths at introns (*ρ* = –0.26 and –0.24, respectively, *P* < 2.2 × 10^–16^) and exons (*ρ* = –0.10 and –0.11, respectively, *P* < 2.2 × 10^–16^). Both forms of methylation exhibit a stronger correlation at the first intron than at all other introns, with the effect being greater for symmetric methylation (*SI Appendix*, Table S3).

Our data reveal a key relationship between asymmetric CpG states and transcription. Thus, we next explored whether this relationship is affected by the orientation of these asymmetric states with respect to a given gene (e.g., whether hemimethylated CpG sites correlate more strongly with transcription when the mC residue is on the coding strand). We observe no appreciable strand-dependent effect of either mC or hmC on transcription (*SI Appendix*, Fig. S8).

We then looked at whether combining information from multiple CpG states can be a more powerful predictor of a gene’s transcription level than individual states. We factored together the levels of CpG states at promoters, introns, or exons in a pairwise manner and determined the resulting correlation with transcription. Of all such combinations, we find that levels of CC at promoters combined with intronic HC/CH correlate most strongly with transcription (*ρ* = 0.54, *P* < 2.2 × 10^–16^)—more than either state does separately, and more than any other CpG state does individually, regardless of region. Together, these data show that different forms of hmC have distinct relationships with transcription.

## Discussion

We have decoded the mESC genome and DNA epigenome simultaneously, resolving the four genetic states (A, C, G, T) and nine epigenetic states of C, mC, and hmC at CpG sites across both strands (CpG states). The question of whether hmC at CpG sites is predominantly symmetric has been controversial and unresolved. Some studies estimated that symmetric hmC predominates in mESCs (33, 34), with relative levels exceeding 90% (33), whereas others have suggested a higher prevalence of asymmetric hmC (15, 35). Our results show that the vast majority (~98%) of hydroxymethylation at CpG sites is asymmetric. Furthermore, our ability to sequence C and mC simultaneously alongside hmC has revealed that both forms of asymmetric hmC (where the CpG site contains hmC and either C or mC on the opposite strand) are relatively common and have distinct genomic distributions and relationships with transcription. This is consistent with previous work using hairpin oxidative bisulfite sequencing, in which different hmC states were statistically inferred at targeted genomic loci in mESCs (36, 37). The authors found substantial levels of both asymmetric forms of hmC (HM/MH and HC/CH) and that these asymmetric forms predominate in both the primed and naïve states (and their intermediates) of the loci examined. The proportion of hmC states was also found to vary according to both locus and mESC state (37). Hairpin-bisulfite sequencing has also been used by others at a wider scale (covering 17.3% of CpG sites in the mouse genome), identifying substantial levels of hemimethylation in mESCs (38), consistent with our work.

Our observation that hmC is overwhelmingly asymmetric genome-wide would preclude a maintenance mechanism for hmC that is analogous to mC. In maintenance methylation, mC symmetry at CpG sites is crucial; upon replication, each daughter strand carries one of the two marks at a given locus, enabling direct transmission of methylation information from the parent cell to both daughter cells. Our findings raise key questions about the fate of hmC upon replication, and how this affects the epigenetic states of daughter cells.

Very recent work using nanopore-based duplex sequencing has found that hmC is also predominantly asymmetric in terminally differentiated tissue (mouse cerebellum) (39). Notably, the relative proportions of hmC states differ markedly between these systems. In the cerebellum, the most abundant states are HM/MH (72% of all hmC), followed by HH (17%) and HC/CH (11%). By contrast, in primed mESCs, we find that the HC/CH states are most abundant (63%), followed by HM/MH (35%) and HH (2%). These observations demonstrate pronounced, tissue-specific differences in the double-stranded mammalian epigenome, which could extend to other cell types. Further work is needed to determine the mechanistic basis of these observations.

We observe key differences in the behavior of hmC, depending on whether C, mC, or hmC occurs on the other strand of the CpG site. Notably, though, for a given asymmetric form of hmC (e.g., HC/CH), we observed no overall strand bias for one orientation versus the other, relative to gene orientation (i.e. whether the hmC is on the sense or antisense strand). This may reflect some equivalence between the two orientations, or perhaps that any differences are resolvable only with longer read lengths or at the single-cell level. Further work is needed to establish this.

Strikingly, we find that total hmC levels at primed enhancers vastly exceed those at poised and active enhancers. Furthermore, poised and active enhancers are somewhat depleted in hmC overall, though with elevated levels of HC/CH at the flanking regions. This seemingly contradicts earlier reports of strong hmC enrichment at these enhancer types (15, 30). However, these studies defined poised enhancers differently by using the presence of H3K4me1 and absence of H3K27ac, rather than the presence of H3K27me3. Such studies may therefore not have differentiated between poised and primed enhancers, as defined by Cruz-Molina et al. (28). For active enhancers, a similar depletion of hmC has indeed been observed by others (30). Our data also reveal the double-stranded context of C, mC, and hmC at enhancers. We observe major differences in the distributions of all CpG states, both within enhancers and between enhancer types. The predominant form of hmC at active, poised and primed enhancers is the HC/CH states. Notably, primed enhancers are unique in also having elevated levels of the HM/MH states, and in having comparatively higher levels of symmetric and hemimethylation, which has not previously been identified. As we cannot determine the lifetime of CpG states, further work is needed to establish whether the abundance of CpG asymmetry at primed enhancers reflects continual cycles of methylation and active demethylation, or whether these marks are stable. However, our finding that Tet-mediated demethylation is low at primed enhancers suggests a distinct role for hmC at this enhancer state. This is consistent with our previous work, showing that a large proportion of hmC in mESCs and other mouse tissue is long-lived (4). At active and poised enhancers, we observe very similar distributions of CpG states, which may reflect that they are alike in many of their features (e.g., p300 and BRG1 binding, and comparable levels of nucleosomal depletion) (40).

As well as being the major form of hmC at enhancers, our results reveal that the HC/CH states largely account for the widely reported correlation (7–10) between gene-body hydroxymethylation and transcription. Notably, the combined levels of intronic HC/CH and promoter CC show a stronger correlation with transcription than either does alone—and strikingly, even more strongly than promoter methylation. This suggests a functional relationship between CpG states in different genic regions and transcription and highlights the importance of simultaneously resolving double-stranded CpG states. Further characterization of this relationship using machine-learning approaches may enhance its predictive power for transcription. Unlike other forms of hmC, the HC/CH states also exhibit clear differences several kilobases either side of the TSS and TTS, where levels are considerably higher even for lowly transcribed genes, indicating that these states mark broader regions of active transcription.

Overall, our work demonstrates that resolving C, mC, or hmC on single-stranded DNA is insufficient to characterize the functional status of a CpG site. The behavior of epigenetic bases also depends on whether C, mC, or hmC occurs on the opposite strand. Thus, different double-stranded forms of hmC are not equivalent, and different combinations of these states at CpG sites constitute distinct units of regulatory information. We anticipate that this methodology will prove useful in providing a more comprehensive analysis of DNA epigenetic states in the future.

## Materials and Methods

### Cell Culture.

Basal medium for mESC cultures was DMEM high glucose (Sigma, D6546-500 mL) containing 15% FBS (Gibco, 16141079), 2 mM GlutaMax-I (Gibco, 35050-038), 1 × NEAA (Gibco, 11140-035) and 0.1 mM β-mercaptoethanol (Sigma, M3148 -25 mL diluted to 50 mM in 50 μM EDTA/PBS solution). mESCs were regularly maintained on gelatin (G9391-100G)-coated plates by supplementing basal medium with 1,000 U/mL LIF (PeproTech, murine LIF, 250-02-25UG). Cultures were routinely monitored for *Mycoplasma* contamination.

### Isolation of gDNA.

Isolation of gDNA samples was performed as described previously (41) with the following modifications: Cultures were washed in PBS and lysed by adding RLT buffer (Qiagen) containing 1:100 β-mercaptoethanol. Isolation of genomic DNA was performed with Zymo-Spin IIC-XL columns according to the instruction of the ZR-Duet DNA/RNA MiniPrep Kit (Zymo Research). Lysates were applied onto spin columns, and bound material was incubated for 10 min with Genomic Lysis Buffer (Zymo Research) supplemented with 0.2 mg/mL RNaseA (Qiagen). After washing genomic DNA was eluted in water.

### Preparation of Sequencing Libraries.

Genomic DNA was sonicated using a Covaris M220 (150-bp target size). Fragments of ~100 bp were then isolated by automated pulsed-field gel electrophoresis (BluePippin, 3% agarose, Sage Science, catalog number BDQ3010) according to the manufacturer’s protocol. 100 ng of the purified fragments was combined with the “H1” and “H2” spike-in controls (0.38 ng each, ATDBio Ltd, see *SI Appendix*, Table S1 for sequences), 1 μL of spike-in control (duet evoC, biomodal, catalog number 6101), and adjusted to 50 μL with low-EDTA TE buffer (VWR, catalog number A8569). Samples were end-repaired and A-tailed (NEB Ultra II End Repair/dA-Tailing Module, catalog number E7546S) and adapter-ligated (NEB Ultra II Ligation Module, catalog number E7595S) according to the manufacturer’s guidelines, except that 3.75 μL of a 1:1 mixture of custom, preannealed hairpin adapters (obtained from ATDBio Ltd, see *SI Appendix*, Table S2 for sequences) was used from a stock of 5 μM per adapter. Prior to their mixing, each of these hairpin adapters was annealed separately in a buffer of 10 mM Tris·HCl, 0.1 mM EDTA, and 100 mM NaCl, pH 8.0.

Following a clean-up (1.6 × SPRISelect, Beckman Coulter, catalog number B23317), eluting in 23.75 μL nuclease-free water, samples were combined with rCutSmart buffer (10×, NEB) and 3.25 μL USER enzyme mix (NEB, catalog number M5505S), incubated (37 °C, 30 min), then purified by spin column (DNA Clean & Concentrator 5, Zymo Research, catalog number D4014), eluting in 13 μL DNA Elution Buffer. 12 μL of each DNA sample was combined with 2.5 μL nuclease-free water, 2.5 μL NEB Buffer 4 (10×), 0.5 μL dNTPs (10 mM stock, NEB, catalog number N0447S), 2.5 μL UDP-glucose (Thermo Scientific, catalog number EO0831), 2 μL Polynucleotide Kinase (NEB, catalog number M0201S), 2 μL Klenow Fragment *exo*– (Thermo Scientific, catalog number EP0422), and 1 μL T4 β-glucosyltransferase (Thermo Scientific, catalog number EO0831). Following incubation (37 °C, 30 min) a 1.2 × SPRISelect clean-up was performed, eluting in 14 μL Tris.HCl (10 mM, pH 8.0). Samples were then denatured and reannealed (95 °C, 2 min, then cooling to 10 °C at a rate of 0.1 °C/s). To ensure maximal ligation efficiency in the following step, 13.5 μL of each reannealed sample was combined with low-TE buffer (11.5 μL), NEBNext Ultra II End Prep Reaction Buffer (3.5 μL) and NEBNext Ultra II End Prep Enzyme Mix (1.5 μL), and incubated (30 min at 20 °C, then 30 min at 65 °C). Ligation of the constructs to an Illumina-compatible Y-shaped adapter was performed by premixing the 30-uL DNA sample with EM-seq adapter (1.25 μL, 15 μM stock, NEB, catalog number E7140S) followed by NEB Ultra II Ligation Master Mix (15 μL) and NEB Ultra II Ligation Enhancer (0.5 μL). Following incubation (20 °C, 15 min), samples were immediately purified (1 × SPRISelect), eluting with 16 μL nuclease-free water. Next, the construct was treated with a series of enzymatic reactions using an early access duet evoC kit from biomodal. The MT Enzyme (2 μL, biomodal) was diluted with nuclease-free water (51.3 μL). To each 15-uL DNA sample was then added MT Buffer (6 μL, biomodal), MT Additive 3 (0.4 μL, biomodal), diluted MT Enzyme (2 μL, biomodal), MT Additive 2 (0.8 μL, biomodal), MT Additive 1 (1.5 μL, biomodal), and nuclease-free water (4.3 μL). Samples were then incubated (23 °C, 1 h). Next, the oxidation reaction was assembled by combining the DNA sample (30 μL) with Ox Additive 1 (10 μL, biomodal, reconstituted according to the manufacturer’s instructions), DTT (1 μL, biomodal), hmCP Enzyme Additive (1 μL, biomodal), hmCP Enzyme (1 μL, biomodal), and Ox Enzyme (2 μL, biomodal). Ox Additive 2 (1 μL, biomodal) was then diluted in nuclease-free water (1,249 μL), and 5 μL of this solution was added to the DNA-containing reaction mixture. Following incubation of the samples (37 °C, 1 h), PRK (1 μL, biomodal) was added, followed by further incubation (10 mins at 37 °C, then 10 mins at 55 °C, lid heated to 85 °C). Samples were then purified (1.8 × SPRISelect); each was eluted in 32 μL nuclease-free water, of which 31 μL was collected. 1 μL of each sample was used for quantitation (Qubit dsDNA HS). For the deamination reaction, to each 30 μL DNA sample was added nuclease-free water (12.7 μL), DA Buffer (17.5 μL, biomodal), ATP (1.8 μL, biomodal), MgCl_2_ (3.5 μL, biomodal), DA Enzyme 1 (2 μL, biomodal), and DA Enzyme 2 (2.5 μL, biomodal) and the samples incubated (90 mins at 37 °C, lid heated to 45 °C). To each sample was added DA Cleanup (6.5 μL, biomodal), followed by SPRI purification (1 × SPRISelect, eluting in 21 μL nuclease-free water). 20 μL of each DNA sample was then combined with Q5U Master Mix (25 μL, NEB, catalog number M0597S) and an EM-seq UDI primer pair (5 μL, NEB, catalog number E7140S), and amplified by 8 cycles of PCR (30 s at 98 °C for initial denaturation, 10 s at 98 °C for denaturation, 30 s at 62 °C for annealing, and 60 s at 65 °C for extension, and 5 min at 65 °C for the final extension). The amplified constructs were purified by a final SPRI clean-up (0.55 × SPRISelect), eluting in 16 μL low-EDTA TE buffer.

### Sequencing.

Libraries were quantified by TapeStation D5000 (Agilent) and diluted to 1.2 to 1.8 nM prior to sequencing. To compensate for the reduced sequence diversity of the deaminated libraries, 10% PhiX (Illumina #FC-110-3001) was added according to the manufacturer’s guidelines. All libraries were sequenced on an Illumina NovaSeq 6000 using the SP Standard workflow with 500-cycle kits in a 251-8-8-251 base-reads setup.

### Sequencing Data Analysis.

#### CpG-state calling from raw reads.

CpG states were extracted by processing raw reads using a custom bioinformatics pipeline [Snakemake v7.3.8 (42)]. First, target reads were filtered (based on their containing the correct configuration of adapter sequences) and trimmed using Cutadapt (v4.6) (43). As different filtering steps were applied to reads 1 and 2, any resulting unpaired reads were removed using SeqKit Pair (v2.7.0) (44). Next, inserts were separated from flanking hairpin-adapter sequences using Cutadapt, such that each read pair produced a set of four insert sequences (a & b from Read 1, c & d from Read 2, Fig. 1*C*). Read pairs that did not yield all four corresponding insert sequences were discarded using SeqKit Pair. The resulting inserts were aligned in paired-end mode (a & d, b & c) using BSBolt (v1.5.0) (45) against the GRCm38 (mm10) reference. Data from lanes 1 and 2 of each NovaSeq run were then merged with SAMtools (v1.19.2) (46) and deduplicated in paired-end mode with Picard (v3.1.1) (47). Next, the paired, deduplicated bam files were split back into the four corresponding insert sequences (a–d) using BamTools (v2.5.2) (48). Methylation calls were extracted at the single-read level using a custom Python (v3.12.1) script. Using a custom *R* (49) script (v4.3), the four methylation calls (from inserts a, b, c, d) associated with each CpG site from each read pair were compiled and used to call the corresponding CpG state (Fig. 1*C*). The genomic coordinates and count of each called CpG state were then compiled. At this stage, data from the two independent experiments were either merged (for the main analysis) or analyzed separately to assess agreement between datasets (*SI Appendix*, Figs. S2–S5). Using a custom *R* script (v4.3.1), CpG sites whose double-stranded depth was below 5× were discarded, as were those which overlapped with blacklisted regions (50) or containing multiple CpG-state calling errors. After filtering, 15.8 million CpG sites were retained. The percentage of each individual CpG state at each CpG site was then calculated, as were total percentages of corresponding asymmetric CpG states (MC + CM, HC + CH, HM + MH). For the analysis comparing the two replicates (*SI Appendix*, Figs. S2–S5), individual processing and filtering of the replicates yielded 13.7 million CpG sites (Replicate 1) and 4.9 million CpG sites (Replicate 2).

#### Confusion matrix.

Data used in the call-rate matrix were derived from all CpG sites of the synthetic spike-ins and analyzed using a custom *R* script (v4.3.1). Further information is given in the Supporting Text.

#### Global analysis.

To infer the true rate of each CpG state in the genome, we used the signal obtained from the oligonucleotide controls to correct the observed levels of each CpG state.

Specifically, each observed CpG state was modeled with a system of 9 linear equations that capture the additive contribution of all the other detected states levels (signal) observed on the oligonucleotide control sequences.

$$O_{i}=\sum_{j=1}^{9}p_{\mathrm{ij}}\times T_{j},$$

where $O_{i}$ is the observed ith CpG state across the genome; $p_{\mathrm{ij}}$ is the combination of all 9 measured CpG states’ levels observed across the oligonucleotide controls; $T_{j}$ is the true level of each CpG state.

With a custom *R* script (v4.3.1), the system of linear equations was instructed. The coefficients $p_{\mathrm{ij}}$ were estimated from the sequencing data as well as the observed genome levels $O_{i}$. The system of linear equations was resolved using the *R* base function *solve*(); any resulting negative values were set to zero. The results obtained from this were visualized in the barplot of Fig. 3*A*.

#### Enhancers.

The enhancer analysis was performed using a custom *R* script (v4.3.1). Briefly, all CpG sites overlapping with enhancer peaks [primed, poised, or active, as defined by Cruz-Molina et al. (28)] were identified with BEDTools (v2.31.0) (51), and mean levels of each CpG state (or grouped, corresponding CpG states) at each enhancer were calculated. This was also done for randomly shuffled enhancer peaks (BEDTools shuffle) to enable the background levels of each state to be determined. Profiles of CpG states at each enhancer type were generated using deepTools (52) (200-bp bin size).

#### Transcription.

RNA-seq data from E14-mESCs cultured in serum/LIF were obtained from a publicly available dataset (53) and, using a custom *R* script (v4.3.1), grouped by transcriptional output (silent, and the tertiles low, medium, and high). CpG coordinates were annotated using ChIPseeker (v1.36.0) (54) (promoter-TSS region defined as 1 kb either side of the TSS) and filtered for protein-coding genes. Mean CpG-state levels were calculated for each gene and annotation (e.g., introns, exons). Spearman’s correlation coefficients were then determined for each CpG state (or grouped corresponded states) for each annotation feature using the *R stats* function *cor.test()* (v4.3.1). Multiple states and annotations were combined by factoring pairwise combinations of CpG-state levels from different annotations. Plots of Fig. 5*C* were produced using deepTools (v3.5.4) (52) from input data binned to 200 bp. For gene-body plots, genes were scaled to 5 kb, and additional binning (100 bp) was applied for clarity. For the TSS/TTS plots, no scaling or additional binning was applied.

## Supplementary Material

Appendix 01 (PDF)

## Data Availability

Sequencing data generated in this study have been submitted to the NCBI Gene Expression Omnibus (GEO; https://www.ncbi.nlm.nih.gov/geo/) under accession number GSE263772 (55). Code developed to process raw sequencing reads and for downstream bioinformatic analysis is available at https://github.com/sblab-informatics/SCoTCH-seq (56). All other data are included in the manuscript and/or *SI Appendix*.

## References

[1] Y. Dor, H. Cedar, Principles of DNA methylation and their implications for biology and medicine. Lancet **392**, 777–786 (2018). 30100054 10.1016/S0140-6736(18)31268-6

[2] M. Tahiliani , Conversion of 5-methylcytosine to 5-hydroxymethylcytosine in mammalian DNA by MLL partner TET1. Science **324**, 930–935 (2009). 19372391 10.1126/science.1170116 PMC2715015

[3] T. Carell, M. Q. Kurz, M. Müller, M. Rossa, F. Spada, Non-canonical bases in the genome: The regulatory information layer in DNA. Angew. Chem. Int. Ed. **57**, 4296–4312 (2018). 10.1002/anie.201708228 28941008

[4] M. Bachman , 5-Hydroxymethylcytosine is a predominantly stable DNA modification. Nat. Chem. **6**, 1049–1055 (2014). 25411882 10.1038/nchem.2064 PMC4382525

[5] R. Yan , Dynamics of DNA hydroxymethylation and methylation during mouse embryonic and germline development. Nat. Genet. **55**, 130–143 (2023). 36539615 10.1038/s41588-022-01258-x

[6] G. P. Pfeifer, W. Xiong, M. A. Hahn, S.-G. Jin, The role of 5-hydroxymethylcytosine in human cancer. Cell Tissue Res. **356**, 631–641 (2014). 24816989 10.1007/s00441-014-1896-7 PMC4090033

[7] B. He , Tissue-specific 5-hydroxymethylcytosine landscape of the human genome. Nat. Commun. **12**, 4249 (2021). 34253716 10.1038/s41467-021-24425-w PMC8275684

[8] X.-L. Cui , A human tissue map of 5-hydroxymethylcytosines exhibits tissue specificity through gene and enhancer modulation. Nat. Commun. **11**, 6161 (2020). 33268789 10.1038/s41467-020-20001-w PMC7710742

[9] I.-H. Lin, Y.-F. Chen, M.-T. Hsu, Correlated 5-hydroxymethylcytosine (5hmC) and gene expression profiles underpin gene and organ-specific epigenetic regulation in adult mouse brain and liver. PLoS One **12**, e0170779 (2017). 28125731 10.1371/journal.pone.0170779 PMC5268415

[10] B. M. Colquitt, W. E. Allen, G. Barnea, S. Lomvardas, Alteration of genic 5-hydroxymethylcytosine patterning in olfactory neurons correlates with changes in gene expression and cell identity. Proc. Natl. Acad. Sci. U.S.A. **110**, 14682–14687 (2013). 23969834 10.1073/pnas.1302759110 PMC3767503

[11] J. Kang , Simultaneous deletion of the methylcytosine oxidases Tet1 and Tet3 increases transcriptome variability in early embryogenesis. Proc. Natl. Acad. Sci. **112**, E4236–E4245 (2015). 26199412 10.1073/pnas.1510510112 PMC4534209

[12] F. Wu , Spurious transcription causing innate immune responses is prevented by 5-hydroxymethylcytosine. Nat. Genet. **55**, 100–111 (2023). 36539616 10.1038/s41588-022-01252-3 PMC9839451

[13] J. N. Kuehner , 5-hydroxymethylcytosine is dynamically regulated during forebrain organoid development and aberrantly altered in Alzheimer’s disease. Cell Rep. **35**, 109042 (2021). 33910000 10.1016/j.celrep.2021.109042 PMC8106871

[14] W. Li , 5-Hydroxymethylcytosine signatures in circulating cell-free DNA as diagnostic biomarkers for human cancers. Cell Res. **27**, 1243–1257 (2017). 28925386 10.1038/cr.2017.121 PMC5630683

[15] M. Yu , Base-resolution analysis of 5-hydroxymethylcytosine in the Mammalian genome. Cell **149**, 1368–1380 (2012). 22608086 10.1016/j.cell.2012.04.027 PMC3589129

[16] P. J. Skene , Neuronal MeCP2 is expressed at near histone-octamer levels and globally alters the chromatin state. Mol. Cell **37**, 457–468 (2010). 20188665 10.1016/j.molcel.2010.01.030 PMC4338610

[17] H. Hashimoto , Recognition and potential mechanisms for replication and erasure of cytosine hydroxymethylation. Nucleic Acids Res. **40**, 4841–4849 (2012). 22362737 10.1093/nar/gks155 PMC3367191

[18] I. E. Wassing , CDCA7 is an evolutionarily conserved hemimethylated DNA sensor in eukaryotes. Sci. Adv. **10**, eadp5753 (2024). 39178260 10.1126/sciadv.adp5753 PMC11343034

[19] A. Shinkai , The C-terminal 4CXXC-type zinc finger domain of CDCA7 recognizes hemimethylated DNA and modulates activities of chromatin remodeling enzyme HELLS. Nucleic Acids Res. **52**, 10194–10219 (2024). 39142653 10.1093/nar/gkae677 PMC11417364

[20] P. Meyer, DNA methylation systems and targets in plants. FEBS Lett. **585**, 2008–2015 (2011). 20727353 10.1016/j.febslet.2010.08.017

[21] X. Wu, Y. Zhang, TET-mediated active DNA demethylation: Mechanism, function and beyond. Nat. Rev. Genet. **18**, 517–534 (2017). 28555658 10.1038/nrg.2017.33

[22] J. Füllgrabe , Simultaneous sequencing of genetic and epigenetic bases in DNA. Nat. Biotechnol. **41**, 1457–1464 (2023). 36747096 10.1038/s41587-022-01652-0 PMC10567558

[23] G. Zheng, X. J. Lu, W. K. Olson, Web 3DNA—A web server for the analysis, reconstruction, and visualization of three-dimensional nucleic-acid structures. Nucleic Acids Res. **37**, 240–246 (2009). 10.1093/nar/gkp358 PMC2703980 19474339

[24] C. D. Laird , Hairpin-bisulfite PCR: Assessing epigenetic methylation patterns on complementary strands of individual DNA molecules. Proc. Natl. Acad. Sci. U.S.A. **101**, 204–209 (2004). 14673087 10.1073/pnas.2536758100 PMC314163

[25] D. Incarnato, A. Krepelova, F. Neri, High-throughput single nucleotide variant discovery in E14 mouse embryonic stem cells provides a new reference genome assembly. Genomics **104**, 121–127 (2014). 25004115 10.1016/j.ygeno.2014.06.007

[26] J. T. Robinson , Integrative genomics viewer. Nat. Biotechnol. **29**, 24–26 (2011). 21221095 10.1038/nbt.1754 PMC3346182

[27] Q. Wang , Pax genes in embryogenesis and oncogenesis. J. Cell. Mol. Med. **12**, 2281–2294 (2008). 18627422 10.1111/j.1582-4934.2008.00427.x PMC4514106

[28] S. Cruz-Molina , PRC2 facilitates the regulatory topology required for poised enhancer function during pluripotent stem cell differentiation. Cell Stem Cell **20**, 689–705.e9 (2017). 28285903 10.1016/j.stem.2017.02.004

[29] S. Heinz, C. E. Romanoski, C. Benner, C. K. Glass, The selection and function of cell type-specific enhancers. Nat. Rev. Mol. Cell Biol. **16**, 144–154 (2015). 25650801 10.1038/nrm3949 PMC4517609

[30] G. C. Hon , 5mC oxidation by Tet2 modulates enhancer activity and timing of transcriptome reprogramming during differentiation. Mol. Cell **56**, 286–297 (2014). 25263596 10.1016/j.molcel.2014.08.026 PMC4319980

[31] C.-W.J. Lio , Tet methylcytosine oxidases: New insights from a decade of research. J. Biosci. **45**, 21 (2020). 31965999 PMC7216820

[32] F. Lu, Y. Liu, L. Jiang, S. Yamaguchi, Y. Zhang, Role of Tet proteins in enhancer activity and telomere elongation. Genes Dev. **28**, 2103–2119 (2014). 25223896 10.1101/gad.248005.114 PMC4180973

[33] Z. Sun , High-resolution enzymatic mapping of genomic 5-hydroxymethylcytosine in mouse embryonic stem cells. Cell Rep. **3**, 567–576 (2013). 23352666 10.1016/j.celrep.2013.01.001 PMC3743234

[34] G. Ficz , Dynamic regulation of 5-hydroxymethylcytosine in mouse ES cells and during differentiation. Nature **473**, 398–404 (2011). 21460836 10.1038/nature10008

[35] C.-X. Song, J. Diao, A. T. Brunger, S. R. Quake, Simultaneous single-molecule epigenetic imaging of DNA methylation and hydroxymethylation. Proc. Natl. Acad. Sci. U.S.A. **113**, 4338–4343 (2016). 27035984 10.1073/pnas.1600223113 PMC4843451

[36] C. Kyriakopoulos, P. Giehr, V. Wolf, H(O)ta: Estimation of DNA methylation and hydroxylation levels and efficiencies from time course data. Bioinformatics **33**, 1733–1734 (2017). 28130236 10.1093/bioinformatics/btx042

[37] P. Giehr , Two are better than one: HPoxBS - hairpin oxidative bisulfite sequencing. Nucleic Acids Res. **46**, e88 (2018). 29912476 10.1093/nar/gky422 PMC6125676

[38] L. Zhao , The dynamics of DNA methylation fidelity during mouse embryonic stem cell self-renewal and differentiation. Genome Res. **24**, 1296–1307 (2014). 24835587 10.1101/gr.163147.113 PMC4120083

[39] D. O. Halliwell, F. Honig, S. Bagby, S. Roy, A. Murrell, Double and single stranded detection of 5-methylcytosine and 5-hydroxymethylcytosine with nanopore sequencing. Commun. Biol. **8**, 243 (2025). 39955446 10.1038/s42003-025-07681-0 PMC11830040

[40] E. Calo, J. Wysocka, Modification of enhancer chromatin: What, how, and why?. Mol. Cell **49**, 825–837 (2013). 23473601 10.1016/j.molcel.2013.01.038 PMC3857148

[41] T. Pfaffeneder , Tet oxidizes thymine to 5-hydroxymethyluracil in mouse embryonic stem cell DNA. Nat. Chem. Biol. **10**, 574–81 (2014). 24838012 10.1038/nchembio.1532

[42] F. Moelder , Sustainable data analysis with snakemake. F1000Res **10**, 33 (2021). 34035898 10.12688/f1000research.29032.1 PMC8114187

[43] M. Martin, Cutadapt removes adapter sequences from high-throughput sequencing reads. EMBnet J. **17**, 10–12 (2011), 10.14806/ej.17.1.200.

[44] W. Shen, S. Le, Y. Li, F. Hu, Seqkit: A cross-platform and ultrafast toolkit for FASTA/Q file manipulation. PLoS One **11**, e0163962 (2016). 27706213 10.1371/journal.pone.0163962 PMC5051824

[45] C. Farrell, M. Thompson, A. Tosevska, A. Oyetunde, M. Pellegrini, BiSulfite Bolt: A bisulfite sequencing analysis platform. GigaScience **10**, giab033 (2021). 33966074 10.1093/gigascience/giab033 PMC8106542

[46] H. Li , The sequence alignment/map format and SAMtools. Bioinformatics **25**, 2078–2079 (2009). 19505943 10.1093/bioinformatics/btp352 PMC2723002

[47] Picard, http://broadinstitute.github.io/picard/.

[48] D. W. Barnett, E. K. Garrison, A. R. Quinlan, M. P. Strömberg, G. T. Marth, BamTools: A C++ API and toolkit for analyzing and managing BAM files. Bioinformatics **27**, 1691–1692 (2011). 21493652 10.1093/bioinformatics/btr174 PMC3106182

[49] R Core Team, R: A Language and Environment for Statistical Computing (R Foundation for Statistical Computing, Vienna, Austria, 2023).

[50] H. M. Amemiya, A. Kundaje, A. P. Boyle, The ENCODE blacklist: Identification of problematic regions of the genome. Sci. Rep. **9**, 9354 (2019). 31249361 10.1038/s41598-019-45839-z PMC6597582

[51] A. R. Quinlan, I. M. Hall, BEDTools: A flexible suite of utilities for comparing genomic features. Bioinformatics **26**, 841–842 (2010). 20110278 10.1093/bioinformatics/btq033 PMC2832824

[52] F. Ramírez , DeepTools2: A next generation web server for deep-sequencing data analysis. Nucleic Acids Res. **44**, W160–W165 (2016). 27079975 10.1093/nar/gkw257 PMC4987876

[53] C. Alda-Catalinas , A single-cell transcriptomics CRISPR-activation screen identifies epigenetic regulators of the zygotic genome activation program. Cell Syst. **11**, 25–41.e9 (2020). 32634384 10.1016/j.cels.2020.06.004 PMC7383230

[54] G. Yu, L.-G. Wang, Q.-Y. He, ChIPseeker: An R/bioconductor package for ChIP peak annotation, comparison and visualization. Bioinformatics **31**, 2382–2383 (2015). 25765347 10.1093/bioinformatics/btv145

[55] J. S. Hardwick, S. Dhir, W. S. Gosal, S. Balasubramanian, SCoTCH-seq reveals that 5-hydroxymethylcytosine encodes regulatory information across DNA strands. Gene Expression Omnibus. https://www.ncbi.nlm.nih.gov/geo/query/acc.cgi?acc=GSE263772. Deposited 11 April 2024. 10.1073/pnas.2512204122 PMC12337322 40743391

[56] J. S. Hardwick, S. Dhir, A. Simeone, SCoTCH-seq code. GitHub. https://github.com/sblab-informatics/SCoTCH-seq. Deposited 15 July 2025.

[57] A. H. Handyside, G. T. O’Neill, M. Jones, M. L. Hooper, Use of BRL-conditioned medium in combination with feeder layers to isolate a diploid embryonal stem cell line. Roux’s Arch. Dev. Biol. **198**, 48–56 (1989). 28305783 10.1007/BF00376370

