# Supplementary material for: SCoTCH-seq reveals that 5-hydroxymethylcytosine encodes regulatory information across DNA strands
Source: Proc Natl Acad Sci U S A. 2025 Jul 31;122(31):e2512204122. doi: 10.1073/pnas.2512204122 (PMC12337322; doi:10.1073/pnas.2512204122)
Supplement: Supplementary file 1 — Appendix 01 (PDF) [file pnas.2512204122.sapp.pdf]

PNAS

Supporting Information for

SCoTCH-seq reveals that 5-hydroxymethylcytosine encodes regulatory information across DNA strands

Jack S. Hardwick[1], Somdutta Dhir[1,2], Angie Kirchner[1,2], Angela Simeone[1,2], Sean M. Flynn[1,2], James M. Edgerton[1], Rafael de Cesaris Araujo Tavares[1,2], Isabel Esaín-García[1,2], David Tannahill[1,2], Paula Golder[3], Jack M. Monahan[3], Walraj S. Gosal[3] and Shankar Balasubramanian[1,2,4*]

1: Yusuf Hamied Department of Chemistry, University of Cambridge, Cambridge CB2 1EW, UK
2: Cancer Research UK Cambridge institute, University of Cambridge, Cambridge CB2 0RE, UK
3: Biomodal, The Trinity Building, Chesterford Research Park, Cambridge CB10 1TS, UK
4: School of Clinical Medicine, University of Cambridge, Cambridge CB2 0SP, UK
[*]To whom correspondence should be addressed.

Email: sb10031@cam.ac.uk

This PDF file includes:

Supporting text
Figures S1 to S8
Tables S1 to S4
SI References

Supporting Text

The following details how the relative orientation of the ground truth with the reference sequence of synthetic spike-ins was accounted for.

For the synthetic spike-ins, there is no physical distinction at the molecular level between corresponding asymmetric states, e.g. 'MH' and 'HM'. Half the time, the same spike-in will go through the library prep in one orientation, and half the time, in the other:

Orientation 1 ('HM'):	Orientation 2 ('MH'):
`Strand A  5'---HG---3'`	`Strand B  5'---MG---3'`
`Strand B  3'---GM---5'`	`Strand A  3'---GH---5'`

The distinction between HM and MH only arises when we create the reference sequence and must assign one strand of a duplex to be the 'plus' strand and the other to be the 'minus'. It is only then that we arbitrarily define whether the ground truth of this CpG site is an 'MH' or 'HM' state.

Once we have made this arbitrary decision, though, the distinction between HM and MH matters. For example, if we choose HM, rather than MH, to be the ground truth, then upon correct alignment to the reference and subsequent CpG-state calling, it will be called as an HM state. If it were called as MH, this would reflect an error and should be counted as such. Furthermore, the HM state may have a different likelihood of being miscalled as, say, HC than CH. This applies to any other asymmetric CpG state, but not to symmetric states: whether we define the ground truth as HM or MH, either state would have the same probability of being called as CC.

Thus, the HM and MH states cannot simply be merged together and averaged, as we would not be able to distinguish correct HM calls from false MH calls, or separate the differing likelihood of HM being called as HC vs CH or other asymmetric states.

In summary, for a synthetic spike-in there is no distinction at the molecular level between HM and MH. The distinction only arises with respect to alignment to the reference sequence. Thus, in the analysis, both of these factors are accounted for; the ground truths are grouped by their two relative orientations with respect to the reference sequence (to reflect the arbitrary choice of the plus strand) and averaged accordingly (to reflect the fact that there is no distinction at the molecular level between the states).

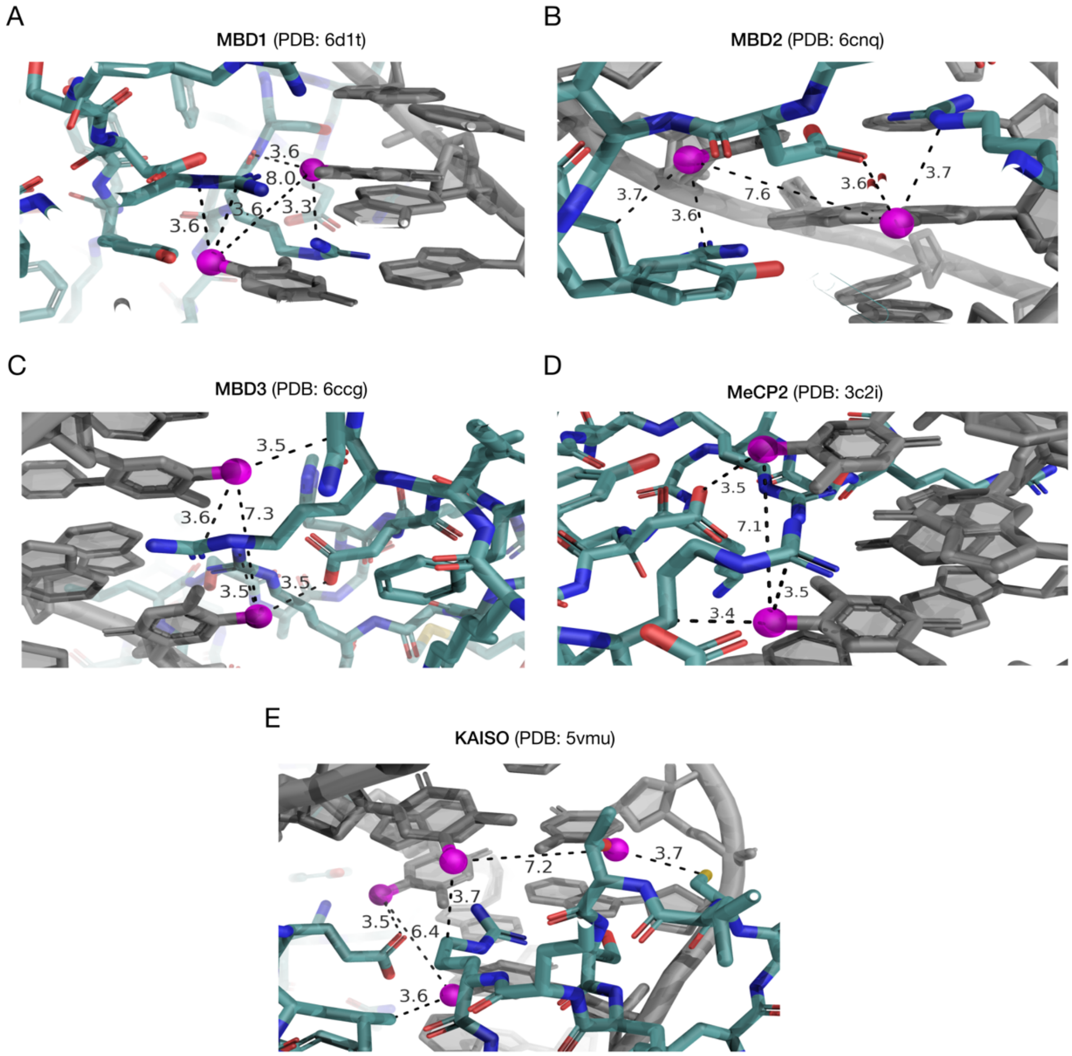

Fig. S1. Crystal structures of known protein readers of modified CpG sites that simultaneously interact with both cytosines. (*A*) MBD1 (PDB: 6d1t), (*B*) MBD2 (PDB: 6cnq (1)), (*C*) MBD3 (PDB: 6ccg (2)), (*D*) MeCP2 (PDB: 3c2i (3)), (*E*) KAISO (PDB: 5vmu (4)), simultaneously interacting with the modifications of both cytosines in each CpG site.

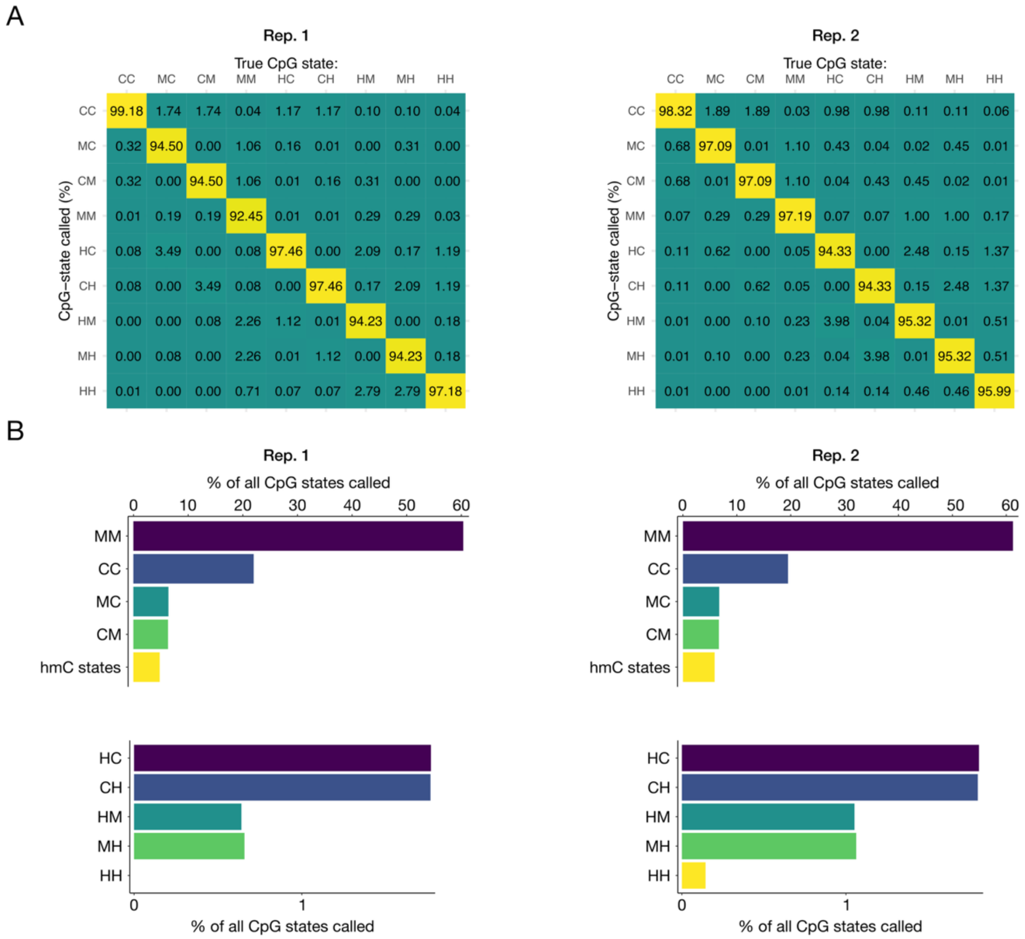

Fig. S2. Comparison of call-rate accuracies and global CpG-state levels for the two replicates. (*A*) Call-rate matrices for the two replicates (Rep. 1: GSM8198270, Rep. 2: GSM8198271). (*B*) Global levels of CpG states for each replicate.

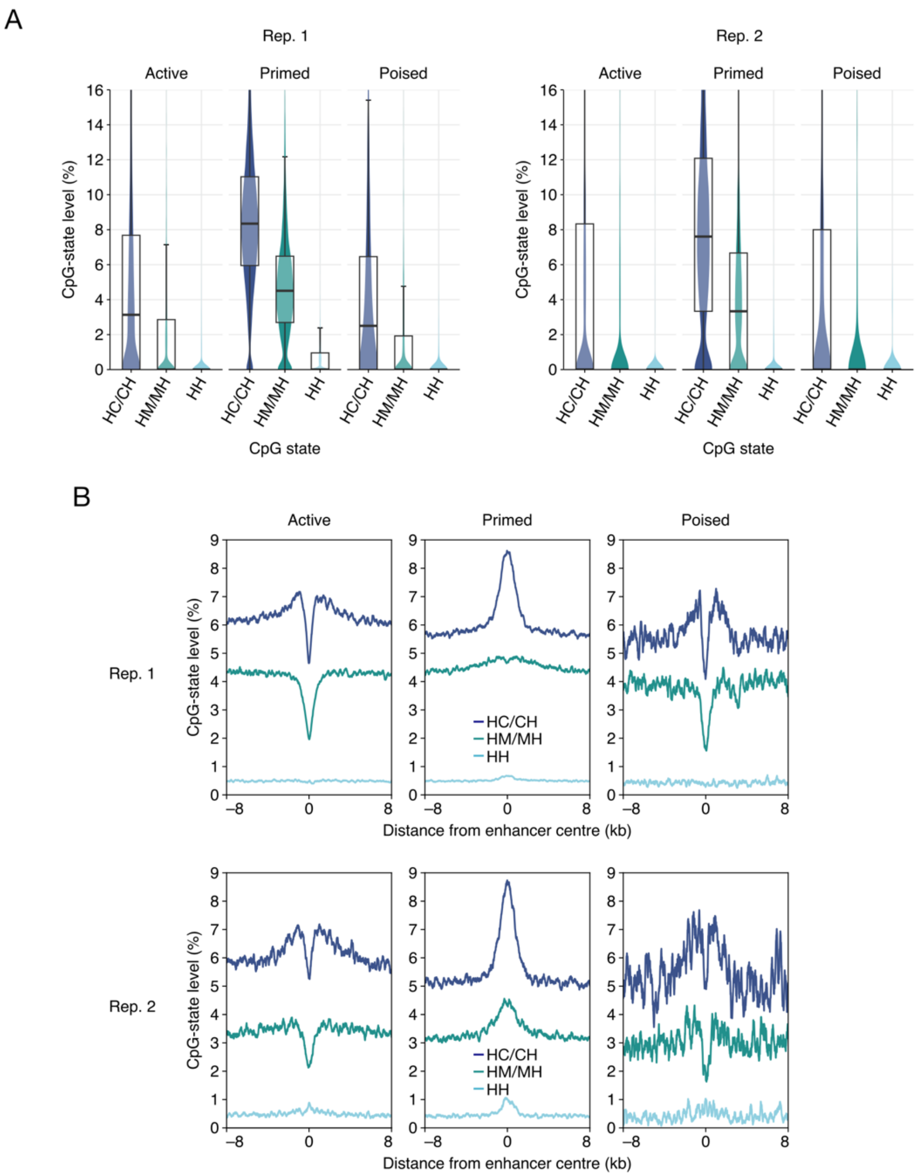

Fig. S3. Comparison of levels and distributions of hmC states at active, primed, and poised enhancers in the two separate, independent sequencing replicates (Rep. 1: GSM8198270, Rep. 2: GSM8198271). (*A*) Average levels of hmC states for each enhancer, in each replicate (centre line, median; box limits, upper and lower quartiles; whiskers, 1.5× interquartile range). (*B*) Density distributions of hmC states in active, primed, and poised enhancers in each replicate.

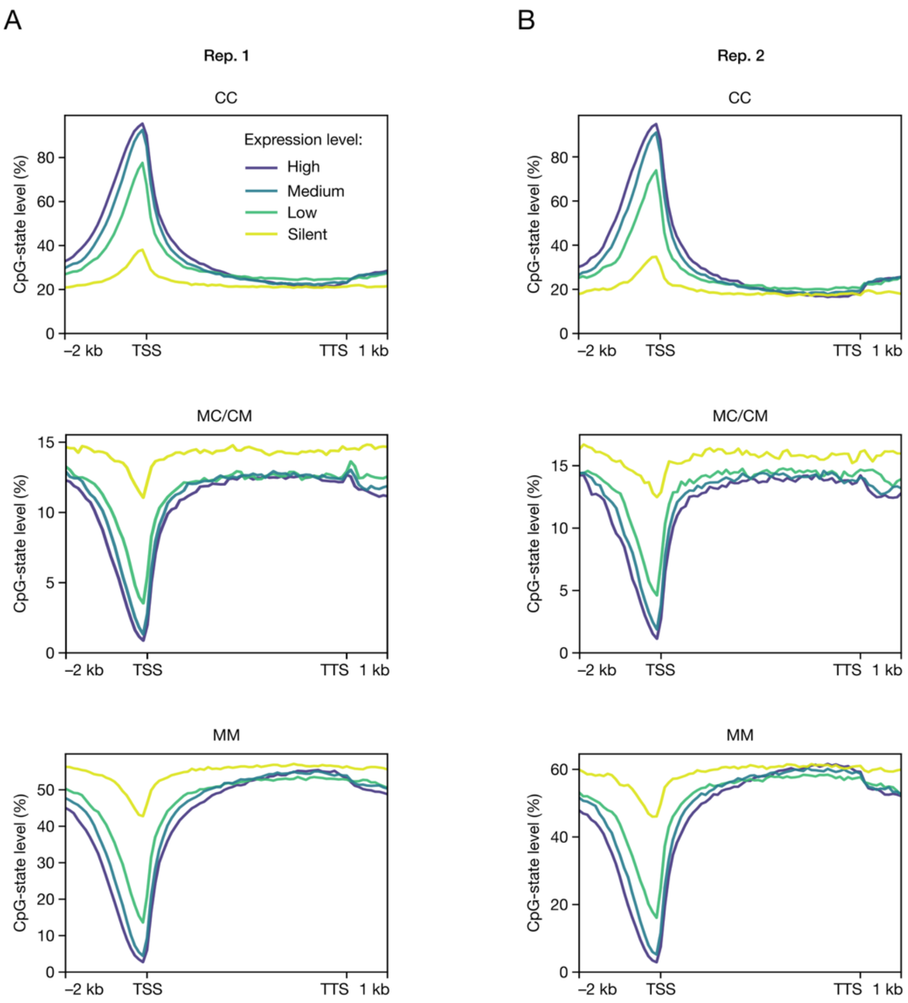

Fig. S4. Density distributions of unmodified (CC), hemimethylated (MC/CM), and symmetrically methylated (MM) CpG states across gene-bodies of protein-coding genes in the two independent replicates (*A*) Rep. 1 (GSM8198270) and (*B*) Rep. 2 (GSM8198271). Genes scaled to 5 kb (regions upstream of TSS and downstream of TTS unscaled).

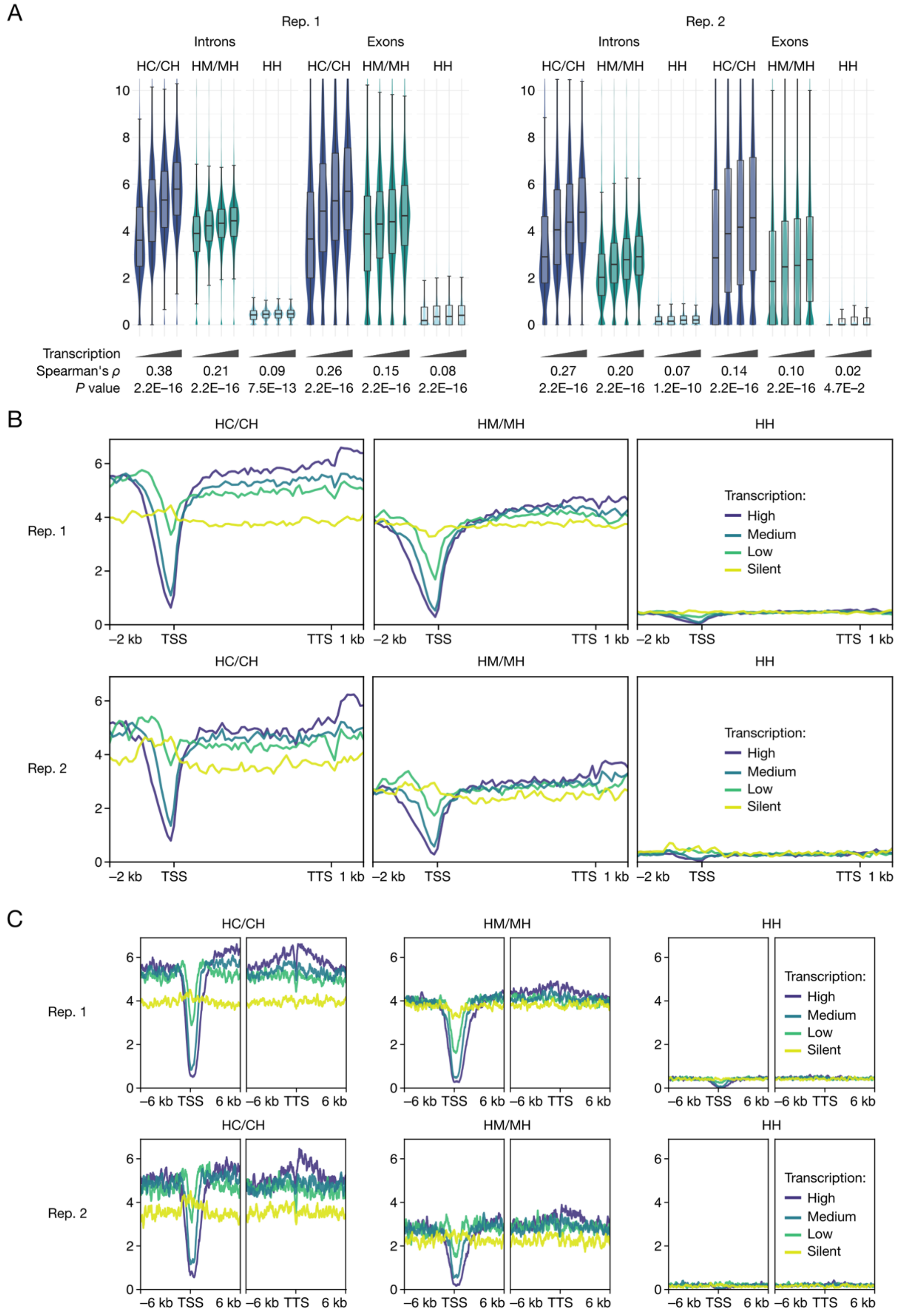

Fig. S5. Comparison of levels and density distributions of hmC states in gene bodies between independent replicates (Rep. 1: GSM8198270, Rep. 2: GSM8198271). (*A*) Levels of hmC states at introns and exons, along with Spearman's correlation coefficients (centre line, median; box

limits, upper and lower quartiles; whiskers, 1.5× interquartile range). (*B*) Density distributions of hmC states across gene bodies (genes scaled to 5 kb) and ±6 kb either side of the TSS and TTS (*C*).

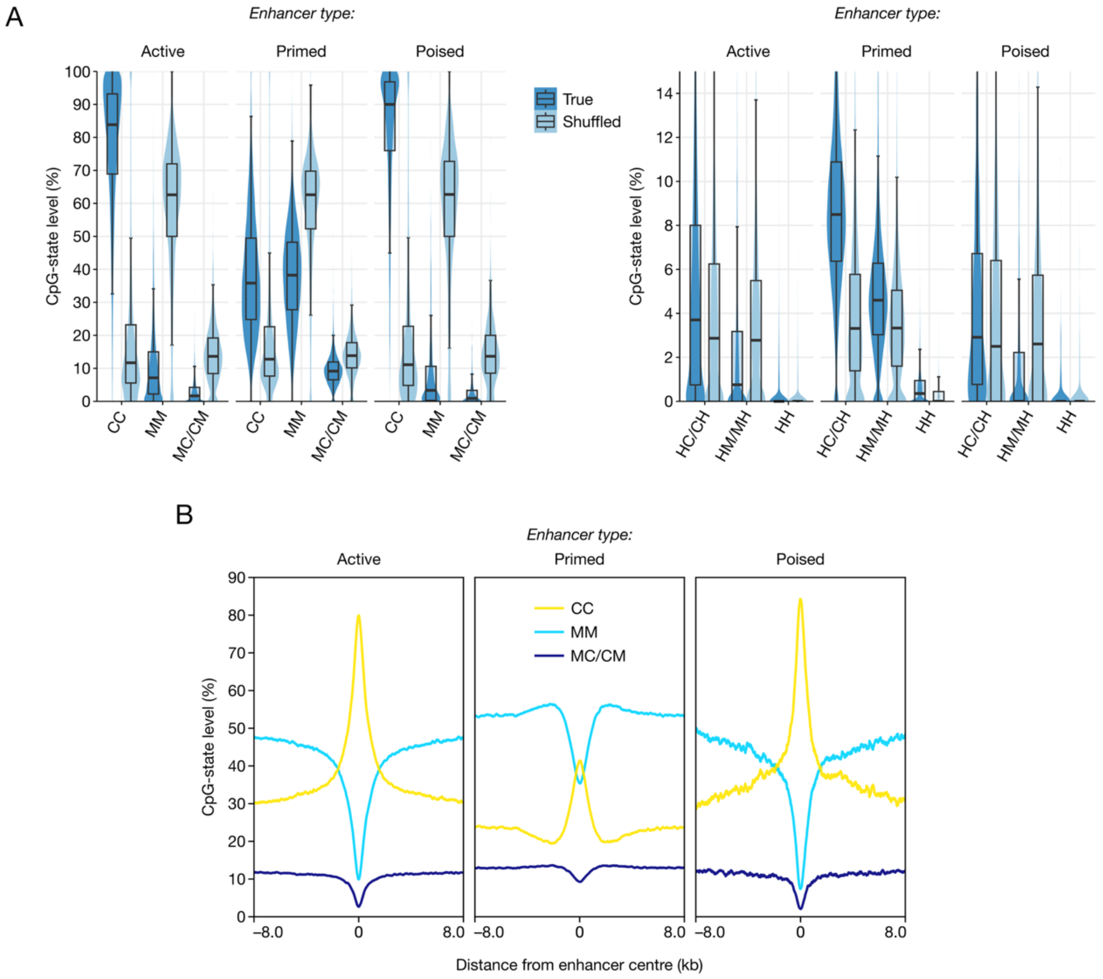

Fig. S6. Levels of all CpG states and density distributions of unmodified (CC), hemimethylated (MC/CM) and symmetrically methylated CpG states at active, primed, and poised enhancers. (*A*) Mean levels of CpG states across each enhancer, grouped by enhancer type (centre line, median; box limits, upper and lower quartiles; whiskers, 1.5× interquartile range). Primed enhancers exhibit distinct levels of all CpG states and are unique in that the majority of CpG sites are modified. (*B*) Density distributions of CC, MC/CM and MM states across active, primed, and poised enhancers. Primed enhancers exhibit unique distributions of CC, MC/CM, and MM states, in addition to the hydroxymethylated states (Fig. 4).

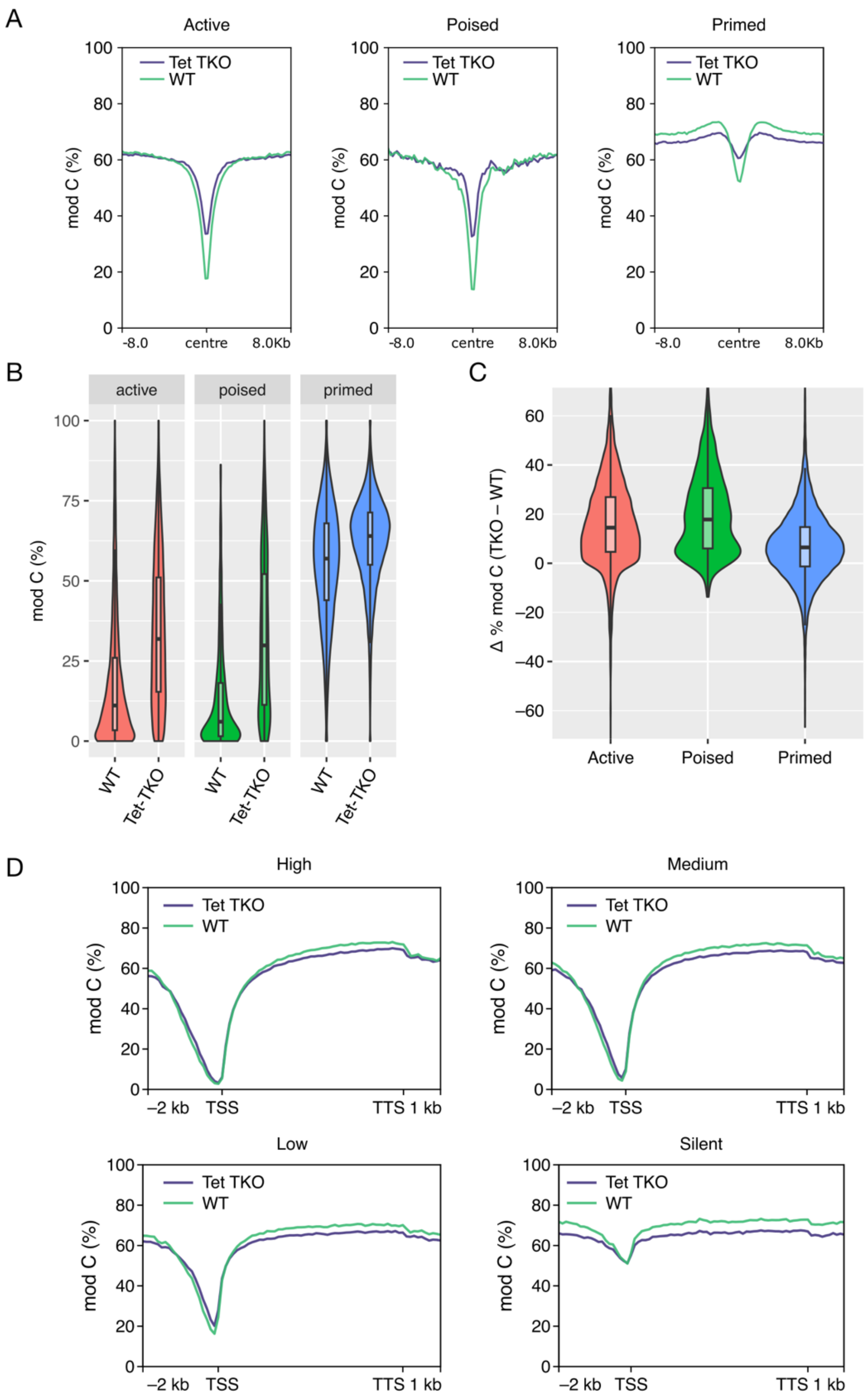

Fig. S7. Comparison of mod C (mC + hmC) levels in Tet-TKO and WT mESCs (reanalysis of data from Lu *et al*,[43], GSE56986). (*A*) Density distributions of mod C at active, poised, and primed enhancers. (*B*) Average mod C levels across active, poised, and primed enhancers. (*C*) Difference between mod C levels in Tet-TKO vs WT mESCs for each enhancer, grouped by enhancer type. Differences are smallest at primed enhancers despite their containing the highest hmC levels, suggesting that Tet-mediated demethylation does not primarily account for the

enrichment of hmC at primed enhancers. (*D*) Density distributions of mod C across bodies of genes grouped by transcript level.

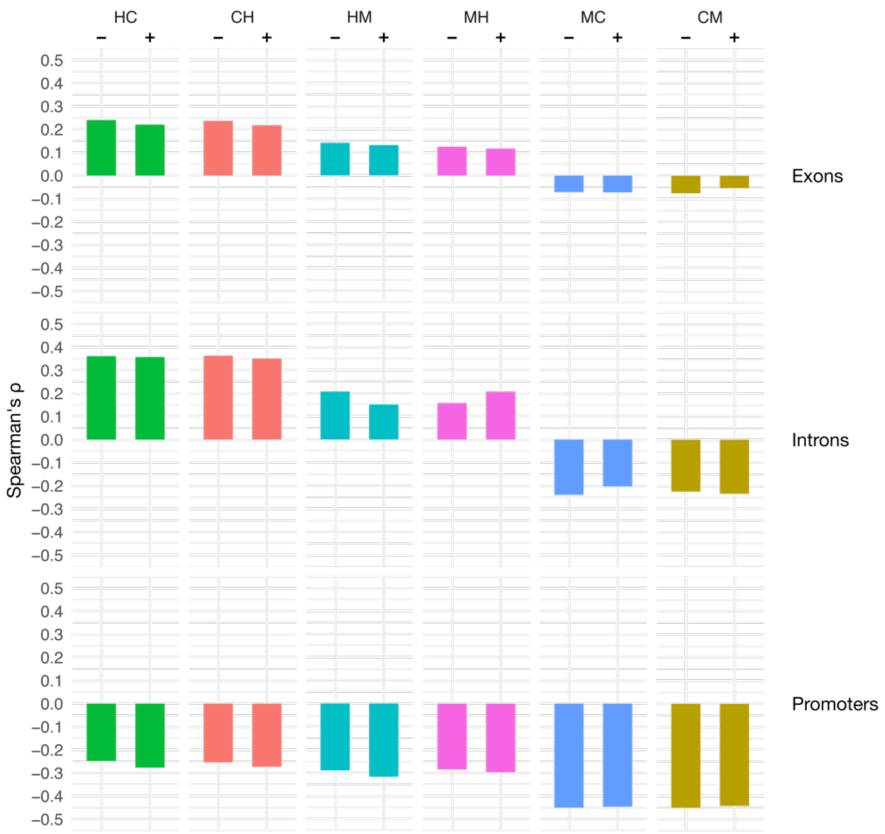

Fig. S8. Spearman's correlations between each asymmetric CpG state (levels averaged across either exons, introns or promoters) and transcription (merged replicates of the double-stranded datasets: GSM8198270 & GSM8198271). Genes are grouped by their orientation, i.e. whether they occur on the plus strand (n = 8,225 genes) or minus strand (n = 8,292 genes). For the HC and CH states, the hmC resides on plus and minus strands, respectively. This is also the case for the HM and MH states. For the MC and CM states, the mC resides on the plus and minus strands, respectively. All P values are $< 2.2 \times 10^{-16}$ except for MC at exons ($P = 2.91 \times 10^{-10}$ and $P = 1.75 \times 10^{-10}$ for genes on the minus and plus strands) and CM at exons ($P = 2.51 \times 10^{-11}$ and $P = 2.77 \times 10^{-6}$ for genes on the minus and plus strands, respectively).

Table S1. Sequences of oligonucleotide spike-ins.

Code	Sequence (5′ to 3′)
H1 (+ strand)	TGACACTATGTT**CG**TCAATACTTCATA**HG**TTGCCTCCACAATG**C**<br>**G**CAATGTAAATGT**MG**CTCAACAAATCCAGTCATAGTTAAGT
H1 (– strand)	ACTTAACTATGACTGGATTTGTTGAG**MG**ACATTTACATTG**CG**CA<br>TTGTGGAGGCAAHGTATGAAGTATTGA**CG**AACATAGTGTCA
H2 (+ strand)	TACCTCCAAATAAGTGAA**CG**GTHGTAA**CG**TTATCCCTGATTGTC<br>TTACTA**CG**ATTAGTA**CG**TATGACACAATT**MG**ACTACAGCTT
H2 (– strand)	AAGCTGTAGT**CG**AATTGTGTCATA**CG**TACTAAT**HG**TAGTAAGAC<br>AATCAGGGATAACGTTA**MG**AC**CG**TTCACTTATTTGGAGGTA
SQC (+ strand)	CAACCACAAATA**CG**ATCA**CG**G**CG**AATC**CG**AT**CG**AATCAGTCAAG<br>**CG**CTTTA**CG**AAGTG**CG**ACAGCCTTAGGGTGGTATGG
SQC (– strand)	CCATACCACCCTAAGGCTGT**CG**CACTTCGTAAAG**CG**CTTGACTG<br>ATT**CG**AT**CG**GATT**CG**C**CG**TGAT**CG**TATTTGTGGTTG
SQ4mC (+ strand)	CTCACTTAACTGCTACTGTT**CG**AGTACTCTGGACTGA**CG**CTTAT<br>TTGGCATTGATTGT**MG**ACAACA**MG**TCCAGAACATCT
SQ4mC (– strand)	AGATGTTCTGGA**MG**TGTTGT**CG**ACAATCAATGCCAAATAAG**CG**T<br>CAGTCCAGAGTACT**MG**AACAGTAGCAGTTAAGTGAG
SQ2hmC (+ strand)	GTATTGACAAGGTGACAGTAT**HG**TCCAGGGACAGTCT**CG**TAGTA<br>CCACCTAGTCTACT**HG**AGAATGTCAAGGTGTCAGAC
SQ2hmC (– strand)	GTCTGACACCTTGACATTCT**CG**AGTAGACTAGGTGGTACTA**CG**A<br>GACTGTCCCTGGA**HG**ATACTGTCACCTTGTCAATAC

M = 5-methyl-2′-deoxycytidine,
H = 5-hydroxymethyl-2′-deoxycytidine, p = phosphate

Table S2. Hairpin adapter sequences.

Code	Sequence (5′ to 3′)
2U hairpin	pATGACGATGCGTT**M**GAGCATCG**U**CA**U**T
2T hairpin	pGTCTGCTACGGTT**M**TACGTAGCAGACT

M = 5-methyl-2′-deoxycytidine, p = phosphate

Table S3. Spearman's rank correlation coefficients between transcription and methylation (symmetric or hemi) of the first intron. $P < 2.2 \times 10^{-16}$ in all cases.

	Spearman's ρ	
	1st intron	Rest of introns
MM	–0.28	–0.12
MC/CM	–0.25	–0.20

Table S4. Sequencing data deposited as part of this work

Accession code	Sample name	Description
GSM8198270	JSH9_E14 replicate 1	Double-stranded sequencing Rep1
GSM8198271	JSH11_E14 replicate 2	Double-stranded sequencing Rep2

SI References

1. K. Liu, *et al.*, Structural basis for the ability of MBD domains to bind methyl-CG and TG sites in DNA. *Journal of Biological Chemistry* **293**, 7344–7354 (2018).

2. K. Liu, *et al.*, Structural analyses reveal that MBD3 is a methylated CG binder. *FEBS Journal* **286**, 3240–3254 (2019).

3. K. L. Ho, *et al.*, MeCP2 Binding to DNA Depends upon Hydration at Methyl-CpG. *Molecular Cell* **29**, 525–531 (2008).

4. E. N. Nikolova, R. L. Stanfield, H. J. Dyson, P. E. Wright, CH···O Hydrogen Bonds Mediate Highly Specific Recognition of Methylated CpG Sites by the Zinc Finger Protein Kaiso. *Biochemistry* **57**, 2109–2120 (2018).
